# Fibro‐inflammatory recovery and type 2 diabetes remission following a low calorie diet but not exercise training: A secondary analysis of the DIASTOLIC randomised controlled trial

**DOI:** 10.1111/dme.14884

**Published:** 2022-06-16

**Authors:** Emer M. Brady, Gaurav S. Gulsin, Evgeny M. Mirkes, Kelly Parke, Prathap Kanagala, Leong L. Ng, Matthew P. M. Graham‐Brown, Lavanya Athithan, Joseph Henson, Emma Redman, Jang Yang, Lei Zhao, Stavroula Argyridou, Laura J. Gray, Thomas Yates, Melanie J. Davies, Gerry P. McCann

**Affiliations:** ^1^ Department of Cardiovascular Sciences University of Leicester and the National Institute for Health Research (NIHR) Leicester Biomedical Research Centre Leicester UK; ^2^ School of Mathematics and Actuarial Science University of Leicester Leicester UK; ^3^ University of Liverpool Liverpool Centre for Cardiovascular Science Liverpool UK; ^4^ Diabetes Research Centre NIHR Leicester Biomedical Research Centre Leicester UK; ^5^ University Hospitals of Leicester NHS Trust Leicester UK; ^6^ Cardiovascular & Fibrosis Translational Research Bristol New Jersey USA; ^7^ Department of Health Sciences University of Leicester Leicester UK

**Keywords:** cardiovascular MRI, diabetic cardiomyopathy, fibrosis, inflammation, type 2 diabetes, type 2 diabetes remission

## Abstract

**Aims:**

To investigate the relationship between fibro‐inflammatory biomarkers and cardiovascular structure/function in people with Type 2 Diabetes (T2D) compared to healthy controls and the effect of two lifestyle interventions in T2D.

**Methods:**

Data were derived from the DIASTOLIC randomised controlled trial (RCT) and includes a comparison between those with T2D and the matched healthy volunteers recruited at baseline. Adults with T2D without cardiovascular disease (CVD) were randomized to a 12‐week intervention either: (1) exercise training, (2) a low‐energy (∼810 kcal/day) meal‐replacement plan (MRP) or (3) standard care. Principal Component and Fisher's linear discriminant analysis were used to investigate the relationships between MRI acquired cardiovascular outcomes and fibro‐inflammatory biomarkers in cases versus controls and pre‐ and post‐intervention in T2D.

**Results:**

At baseline, 83 people with T2D (mean age 50.5 ± 6.4; 58% male) and 36 healthy controls (mean age 48.6 ± 6.2; 53% male) were compared and 76 people with T2D completed the RCT for pre‐ post‐analysis. Compared to healthy controls, subjects with T2D had adverse cardiovascular remodelling and a fibro‐inflammatory profile (20 differentially expressed biomarkers). The 3D data visualisations showed almost complete separation between healthy controls and those with T2D, and a marked shift towards healthy controls following the MRP (15 biomarkers significantly changed) but not exercise training.

**Conclusions:**

Fibro‐inflammatory pathways and cardiovascular structure/function are adversely altered before the onset of symptomatic CVD in middle‐aged adults with T2D. The MRP improved the fibro‐inflammatory profile of people with T2D towards a more healthy status. Long‐term studies are required to assess whether these changes lead to continued reverse cardiac remodelling and prevent CVD.

**NOVELTY STATEMENT**

**What is already known?**

- Diabetic cardiomyopathy is a distinct clinical entity with complex poorly understood pathobiology and limited effective treatment options.
- Inflammation leading to interstitial fibrosis is postulated as a mechanism involved in the development of diabetic cardiomyopathy, but more research is required particularly early in the disease course.

**What this study has found?**

- Evidence of alterations in fibro‐inflammatory pathways and cardiovascular structure/function before the onset of symptomatic cardiovascular disease (CVD) in people with Type 2 Diabetes (T2D).
- A marked beneficial impact of a low‐energy meal replacement plan compared to an exercise training intervention on circulating plasma fibro‐inflammatory biomarkers and cardiovascular structure and function.

**What are the clinical implications of the study?**

- This study provides evidence of additional benefit of a low‐energy meal replacement plan beyond weight reduction and remission of diabetes.
- Long‐term studies are required to assess whether these changes reduce the risk of incident CVD.

## 1 INTRODUCTION

The excess risk of heart failure for patients with Type 2 diabetes (T2D) is estimated to be 45% higher, compared to age‐ and sex‐matched controls.^1^ Numerous pathological changes have been described which predispose those with T2D to heart failure and several cardiovascular abnormalities can be detected on imaging before symptom onset.^2^ When patients become symptomatic, there is a predisposition towards development of heart failure with preserved ejection fraction (HFpEF), which currently has limited proven effective treatments and is associated with adverse outcomes.^3^ Heart failure in relation to T2D is thought to be driven, at least in part, by the upregulation of fibrotic and inflammatory pathways from the metabolic insults of obesity and dysglyceamia.^2^ These drive the development of interstitial myocardial fibrosis, resulting in deterioration of cardiac function.^4^

We have previously shown in a cohort of patients with HFpEF, that those with T2D had significantly greater concentric left ventricular remodelling and elevation of several circulating fibro‐inflammatory markers compared to those without diabetes.^5^ Whether these relationships exist in patients with T2D before the onset of HFpEF is not known.^6^ The identification of novel therapeutic targets early in the natural history is paramount for the development of preventative strategies for heart failure in this population.

The aims of this study were to examine the fibro‐inflammatory profiles of patients with T2D free of cardiovascular disease (CVD) compared to matched healthy controls and the response to exercise training or a low‐energy meal replacement plan (MRP) in subjects with T2D.^7^

## 2 PARTICIPANTS AND METHODS

**FIGURE 1:**
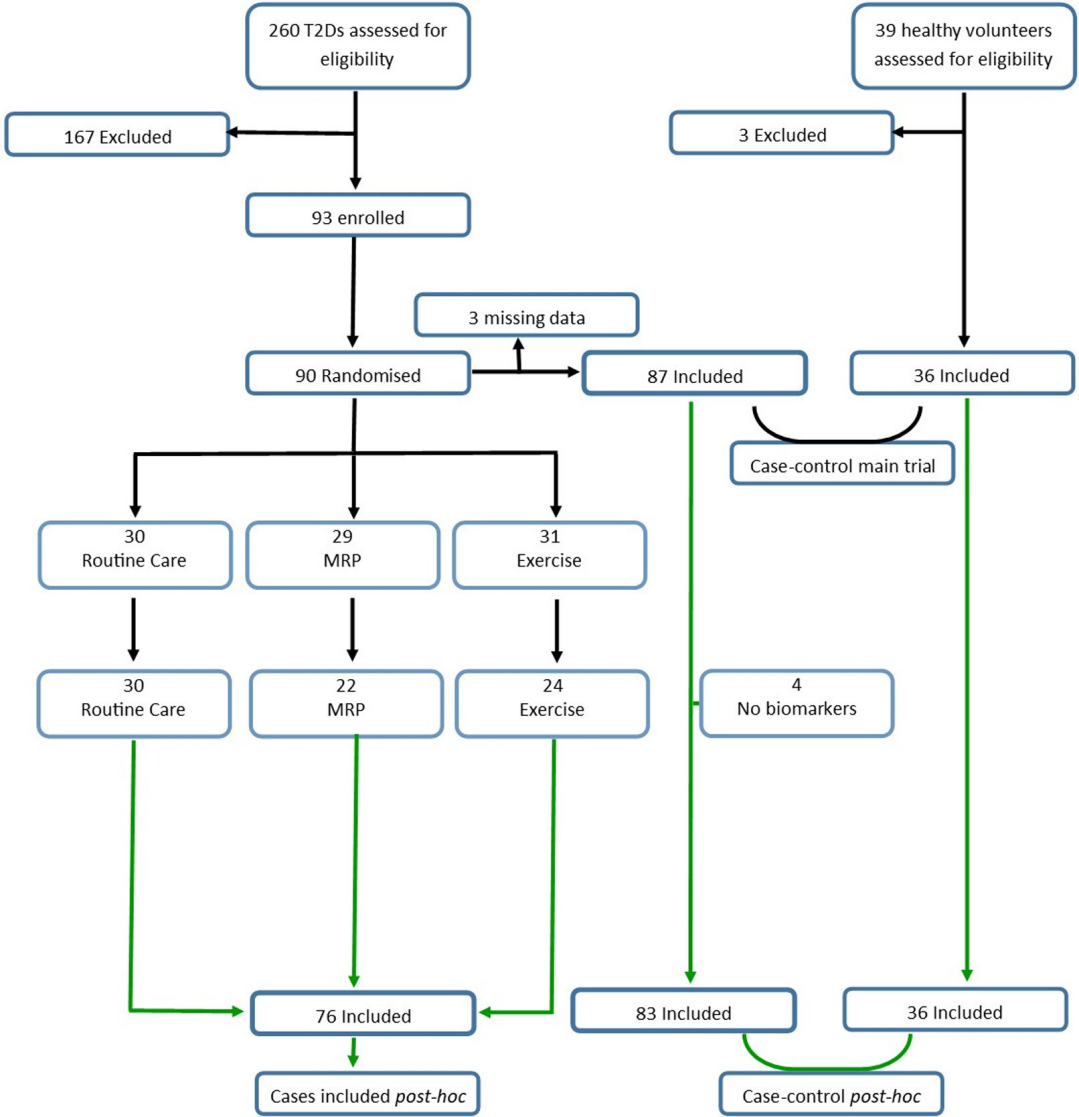
Consort diagram.

This is a post hoc analysis of data from the Diabetes Interventional Assessment of Slimming or Training tO Lessen Inconspicuous Cardiovascular Dysfunction (DIASTOLIC) study.^7^ DIASTOLIC was a prospective, randomised, open‐label, blinded end‐point trial that assessed the effects of a 12‐week intervention with either: thrice weekly supervised exercise training, a low‐energy MRP or routine care on sub‐clinical cardiac dysfunction in adults with T2D without signs, symptoms or evidence of prevalent CVD. The study protocol and the trial primary results have been previously published.^7^, ^8^ Detailed inclusion/exclusion criteria, description of randomisation and the interventions are provided in supplementary material 1.1–3. At baseline, age‐, sex‐ and ethnicity‐matched healthy controls were enrolled for the case–control comparison. The study is registered with www.clinicaltrials.gov (NCT02590822) and has National Research Ethics Service approval (15/WM/0222). All participants provided informed consent prior to any data collection. A study consort diagram is shown in Figure 1.

### 2.1 Plasma biomarkers

Participants fasted overnight and took their morning medication(s) after the study visit. Their last meal was not standardised. Fasting blood samples were obtained and the residual supernatant plasma stored at −80°C prior to batch analysis. HbA1c, glucose, liver, kidney function and lipid profile were analysed according to standard operating procedures in the accredited laboratory at University Hospitals Leicester NHS Trust.

Insulin and leptin were quantified by multiplex assay on a Luminex platform, as previously described.^8^ Plasma Adiponectin was analysed using a Quantikine ELISA assay (R&D Systems, Minneapolis, USA). All samples were assayed in duplicate (the mean reported), with all samples having a co‐efficient of variance (%CV) ≤ 20%.

The Luminex multiplexed plasma biomarker assay (Bristol Myers Squibb, Ewing Township, New Jersey, USA) quantifies multiple biomarkers in a single plasma sample. Each sample was analysed in duplicate with the mean reported and repeated where the CV exceeded 30%. The panel includes markers reflecting inflammation, fibrosis, myocardial injury, reactive oxygen species. Luminex® Assays and Luminex High Performance Assays: R&D Systems. 2017 (https://www.rndsystems.com/products/luminex‐assays‐and‐high‐performance‐assays).The full list of biomarkers of interest is provided in supplemental material 1.3.

### 2.2 Demographics and anthropometrics

Demographics, medical history, and anthropometric measures were collected as described.^8^ Total fat mass, visceral fat and fat‐free mass were assessed by a dual‐energy X‐ray absorptiometry scan (Lunar iDEXA, General Electric Healthcare, Bedford, UK).

### 2.3 Cardiovascular MRI

MRI scans were conducted on a 1.5 T platform (MAGNETOM Aera; Siemens, Erlangen, Germany) as previously described.^8^ Images were analysed offline blinded to treatment group. The MRI outcomes of interest (supplementary material 1.4) were selected to permit investigation of cardiovascular structure and function.

### 2.4 Transthoracic echocardiography

Echocardiography was performed and interpreted by two accredited operators using an iE33 system with S5‐1 transducer (Philips Medical Systems, Best, the Netherlands) to estimate Left Ventricular (LV) filling pressures (E/e').

### 2.5 Statistical analysis

Data were assessed for normality using histograms and Q‐Q plots to permit selection of the appropriate statistical test based on the distribution of each variable. All plasma biomarkers were log10 transformed. Continuous data were reported as mean (± standard deviation [SD]) if normally distributed or median (interquartile range) for non‐normally distributed data, categorical data reported as count (percentage).

### 2.6 Case–control analysis

Cases (T2D) and healthy controls (controls) were compared using independent t‐tests, Mann–Whitney as appropriate, with Chi‐Square tests for categorical variables. Pearson's correlations were performed for all biomarkers and cardiovascular MRI measures for cases and controls.

The total dataset for case–control analysis includes key demographic (age, sex, ethnicity, smoking status) and anthropometric data (body mass index [BMI], and weight and body composition), MRI outcomes, fibro‐inflammatory panel and indices of glycaemic control totalling 75 variables. Principal component analysis with visualisation was utilised to determine if the relationship between the fibro‐inflammatory biomarkers and measures of cardiovascular structure/function were different between the cases and controls with inclusion of the key demographic variables. The principal components were calculated using standardised variables (subtraction of the mean and dividing by SD) and the first three eigenvalues of the covariance matrix by the singular value decomposition method.^9^ The participants were then plotted across this space, calculating the plane with best‐fit through the system of points.^10^

### 2.7 Pre‐ and post‐intervention analysis

The primary focus of the pre‐post intervention analysis was to assess the impact of the 12 week exercise training and MRP interventions in the randomised controlled trial. Repeated measures analysis of variance (ANOVA), with Bonferroni correction applied (observed uncorrected p‐value multiplied by the number of comparisons), was used for the pre‐post‐RCT changes in fibro‐inflammatory biomarkers (Model 1 unadjusted, Model 2 adjusted for change in weight and baseline weight, Model 3 adjusted for change in HbA1c and baseline HbA1c). Cardiac volumes and Left Ventricular mass were indexed for body surface area for the pre‐post analysis preventing overestimating change and absolute values were used for case–control comparison as per standard practice.

To determine whether there was a response to the MRP or exercise training intervention and to assess whether post‐intervention in either group represented a phenotype closer to the healthy controls, Fisher's linear discrimination was used. The data included; fibro‐inflammatory markers, MRI measures, body composition and glycaemic indices. Variables were standardised and the central point of each of the three groups calculated. The vertical axis direction was defined as the vector from the centre of healthy control group to the centre of pre‐intervention group minus projection of them onto the horizontal axis. Both axis vectors were normalised to unit length. Projections of centres of groups of participants pre‐ and post‐intervention are graphically presented with Fisher's discriminant for separation of groups pre‐ and post‐intervention for the points in plane under consideration.^11^ The mathematical process utilised is provided in supplementary material 1.5. K‐nearest neighbour (kNN) based data imputation function (kNNImpute) was used for missing data (open source Data Imputation software [Table S5]).

Data were analysed using IBM SPSS Statistics for Windows, Version 26.0. Armonk, NY: IBM Corp, Python 3.7 (SciPy 1.1.0) and MATLAB (R2018a). Data and statistics software can be found at: https://github.com/Mirkes/Fibro‐inflammatory‐paper.

## 3 RESULTS

### 3.1 Case–control

Ninety cases were recruited to and randomised within the RCT (Figure 1). A total of 83 subjects with T2D and 36 healthy controls had biomarker data and were included in the case–control analysis (Table 1). Those with T2D had greater weight, BMI, blood pressure and were more likely to have smoked. There was evidence of concentric LV remodelling (increased LV mass/volume), smaller LV end diastolic volume and maximal indexed left atrial volume, higher LV filling pressures (E/e’), lower myocardial perfusion reserve and lower aortic distensibility in cases compared to controls. Overall, renal function was preserved and similar between cases and controls.

**TABLE 1:** Participant demographics and cardiovascular structure and function

Variable	Healthy controls (*n* = 36)	T2D (*n* = 83)	*p*‐value
Age (years)	48.6 ± 6.2	50.5 ± 6.4	0.159
Sex (male [%])^b^	19 (53)	48 (58)	0.610
Ethnicity (White European [%])^b^	24 (67)	50 (60)	0.507
T2DM duration (months)^a^	NA	62.4 (38.2)	NA
Smoking history^b^	9 (25)	39 (47)	**<0**.**001**
Hypertension (yes)^b^	0 (0)	43 (52)	**<0**.**001**
HbA1c (mmol/mol)	35.3 ± 2.6	55.9 ± 11.2	**<0**.**001**
HbA1c (%)	5.4 ± 0.2	7.3 ± 1.0	**<0**.**001**
Fasting glucose	5.0 ± 0.5	8.4 ± 2.4	**<0**.**001**
eGFR^a^ (mL/min/1.73m^2^)	85.0 (13.4)	83.9 (9.6)	0.122
Urea (mmol/L)	5.3 ± 1.4	5.4 ± 1.2	0.590
Creatinine (mmol/L)	78.6 ± 1 1.8	75.5 ± 15.3	0.332
Weight (Kg)	70.4 ± 10.8	102.4 ± 16.0	**<0**.**001**
BMI (kgm^2^)	24.5 ± 2.4	36.4 ± 5.4	**<0**.**001**
Resting SBP	120.9 ± 13.2	139.9 ± 15.6	**<0**.**001**
Resting DBP	76.4 ± 7.2	87.6 ± 8.0	**<0**.**001**
Cardiac structure and function			
Indexed LV end‐diastolic volume (mL/m^2^)	83.1 ± 18.6	67.4 ± 10.6	**<0.001**
LV ejection fraction (%)	65.2 ± 4.9	68.20 ± 6.9	**0**.**019**
Indexed LV mass (g/m^2^)	58.1 ± 13.6	55.2 ± 9.0	0.164
LV mass:volume (g/mL)^a^	0.70 (0.1)	0.83 (0.2)	**<0**.**001**
LV global longitudinal strain (+%)	17.6 ± 1.5	16.9 ± 2.6	0.203
LV global gircumferential strain (+%)	19.7 ± 1.9	20.3 ± 2.5	0.190
LV longitudinal peak early diastolic strain rate (s^−1^)	0.86 ± 0.15	0.83 ± 0.13	0.221
LV circumferential peak early diastolic strain rate (s^−1^)	1.01 ± 0.19	1.09 ± 0.16	**0**.**020**
Indexed left atrial maximal volume (mL/m^2^)^a^	50.2 (18.1)	30.9 (9.4)	**<0**.**001**
Left atrial ejection fraction (%)	59.0 ± 7.2	57.1 ± 8.4	0.231
Mean aortic distensibility (mmHg^−1^ × 10^−3^)	6.6 (2.0)	4.2(2.1)	**<0**.**001**
Myocardial perfusion reserve	4.0 ± 1.0	3.1 ± 1.0	**<0**.**001**
Late gadolinium enhancement (%)	2.0 (5.6%)	4.0 (4.8%)	0.855
Average E/e'^a^	6.2 (2.6)	8.0 (3.3)	**0**.**006**

*Note*: Abbreviations: LV = Left ventricular; Indexed measurements are by body surface area; NA = Not applicable.

Data are reported as mean ± standard deviation, count (percent) or median (interquartile range). Bold font highlights statistically significant difference with significance level of 95%.

^a^ Mann–Whitney U test data not normally distributed.

^b^ Results from Chi square test.

### 3.2 Fibro‐inflammatory biomarker profiles

There were significant differences in 20 biomarkers of fibrosis, myocardial injury, atrial stretch, myocardial hypertrophy, inflammation and oxidative stress, renal markers and endothelial dysfunction between healthy controls and T2D (Table 2). The correlation matrices for T2D and healthy controls are presented in Tables S1–S4. Only five correlates were similar across the two groups for only two biomarkers.

**TABLE 2:** Cohort characteristics by group for fibro‐inflammatory biomarkers

Biomarker	Healthy controls (*n* = 36)		T2D (*n* = 83)		*p‐*value
Interstitial fibrosis	Log10	Back transformed*	Log10	Back transformed*	
Galectin3	3.79 ± 0.17	6165.95 ± 1.48	3.74 ± 0.13	5495.41 ± 1.35	**0.052**
MMP2	5.49 ± 0.13	309029.54 ± 1.35	5.40 ± 0.10	251,189 ± 1.26	**<0.001**
MMP3	4.09 ± 0.23	12302.69 ± 1.70	3.93 ± 0.22	8511.38 ± 1.66	**0.001**
MMP7	3.33 ± 0.26	2137.96 ± 1.82	3.52 ± 0.27	3311.31 ± 1.86	**<0.001**
MMP8	2.09 ± 0.35	123.03 ± 2.24	2.28 ± 0.33	190.55 ± 2.14	**0.007**
PAI1	4.87 ± 0.26	74131.02 ± 1.82	5.00 ± 0.17	100000.00 ± 1.48	**0.001**
Tenascin C	4.20 ± 0.19	15848.93 ± 1.55	4.04 ± 0.17	10964.80 ± 1.48	**<0.001**
Myocardial injury					
Troponin T	1.75 ± 0.23	56.23 ± 1.70	1.64 ± 0.21	43.65 ± 1.62	**0.014**
Atrial stress/stretch					
NTproANP	3.69 ± 0.39	4897.79 ± 2.46	3.46 ± 0.31	2884.03 ± 2.04	**0.001**
Myocardial hypertrophy					
Renin	2.97 ± 0.50	933.25 ± 3.16	3.15 ± 0.43	1412.54 ± 2.69	
Inflammation/oxidative stress					
Pentraxin 3	2.13 ± 0.42	134.90 ± 2.63	1.94 ± 0.46	87.10 ± 2.88	
GDF15	2.68 ± 0.33	478.63 ± 2.14	2.97 ± 0.25	933.25 ± 1.78	**<0.001**
Adiponectin	3.91 ± 0.31	8128.31 ± 2.04	3.58 ± 0.24	3801.89 ± 1.74	**<0.001**
Leptin	3.65 ± 0.42	4466.84 ± 2.63	4.29 ± 0.35	19498.4 ± 2.24	**<0.001**
FABP4	4.56 ± 0.36	36307.81 ± 2.29	4.88 ± 0.22	75857.80 ± 1.66	**<0.001**
IL6	0.05 ± 0.05	1.12 ± 1.12	0.12 ± 0.12	1.32 ± 1.32	**0.001**
TNFR1	3.40 ± 0.118	2511.89 ± 1.31	3.49 ± 0.16	3090.30 ± 1.45	**0.011**
TNFR2	5.04 ± 0.25	109647.82 ± 1.78	4.92 ± 0.24	83176.4 ± 1.74	**0.016**
TNF alpha	0.40 ± 0.21	2.51 ± 1.62	0.52 ± 0.21	3.31 ± 1.62	**0.005**
Renal marker					
NGAL	5.50 ± 0.16	316227.77 ± 1.45	5.40 ± 0.16	251,189 ± 1.45	**0.030**
Cystatin C	6.62 ± 0.23	4168693.84 ± 1.70	6.48 ± 0.23	3019952.00 ± 1.70	
Endothelial dysfunction					
VEGFR1	1.25 ± 0.27	17.78 ± 1.86	1.11 ± 0.20	12.88 ± 1.58	**0.001**
VEGFa	1.42 ± 0.18	26.30 ± 1.51	1.53 ± 0.15	33.88 ± 1.41	**0.002**

*Note*: Data reported as mean ± standard deviation with values reported as Log10 and corresponding back transformations (pg/ml). Results only reported for those biomarkers that were statistically different between the two group (at the level *p* < 0.05 and *p* < 0.01).

Bold font highlights statistically significant difference with significance level of 95%.

### 3.3 Case–control visualisation

**FIGURE 2:**
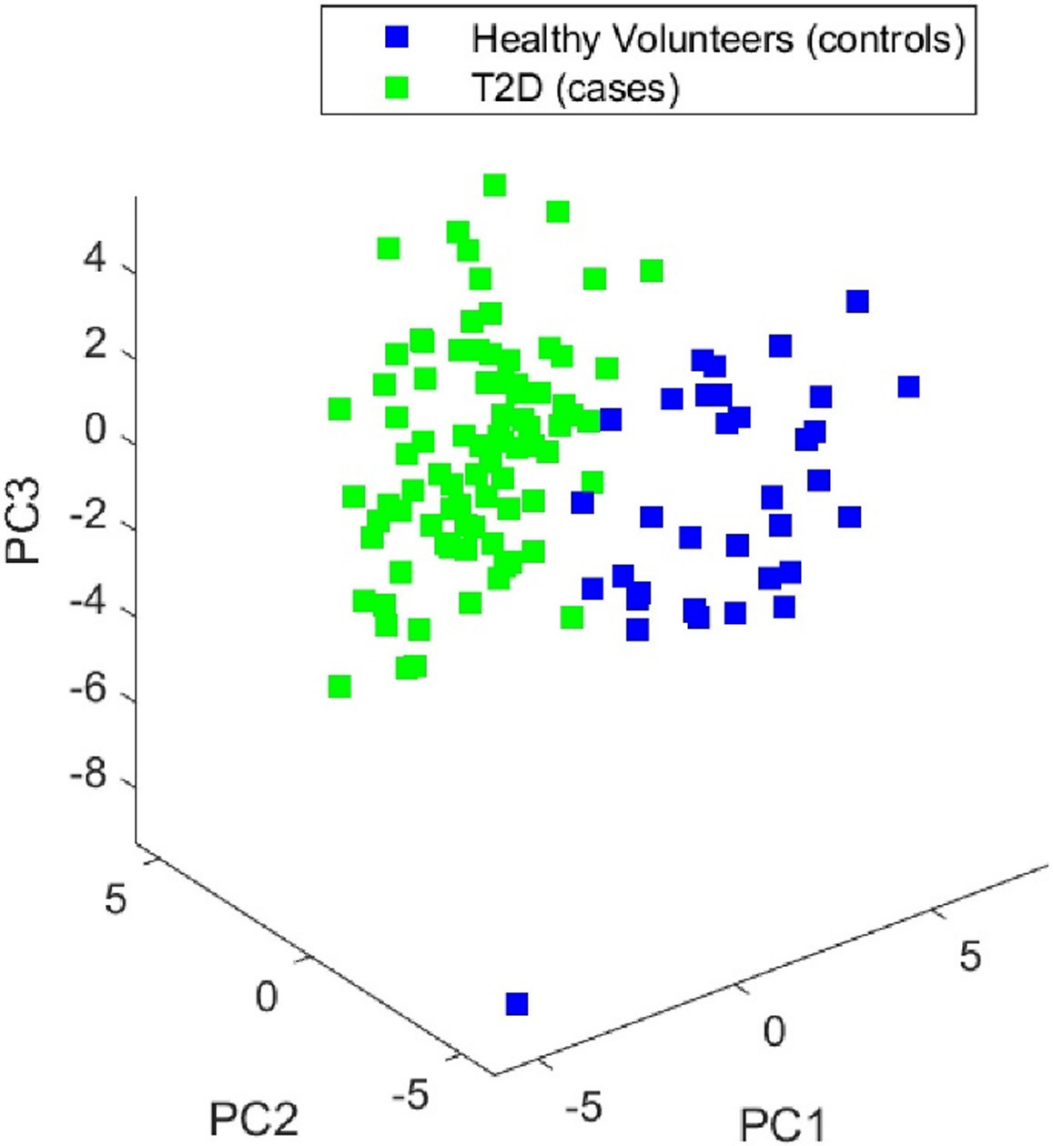
Principal components and 3D‐visualisation for cases and control.

The principal component visualisation is presented in Figure 2. There was almost complete separation of the cases and controls. Variances explained by PCs are PC1 13.60%, PC2 9.71% PC3 7.62%. The separation remains evident when restricted to either the MRI outcomes or fibro‐inflammatory biomarkers (Figures S2–S3).

**FIGURE 3:**
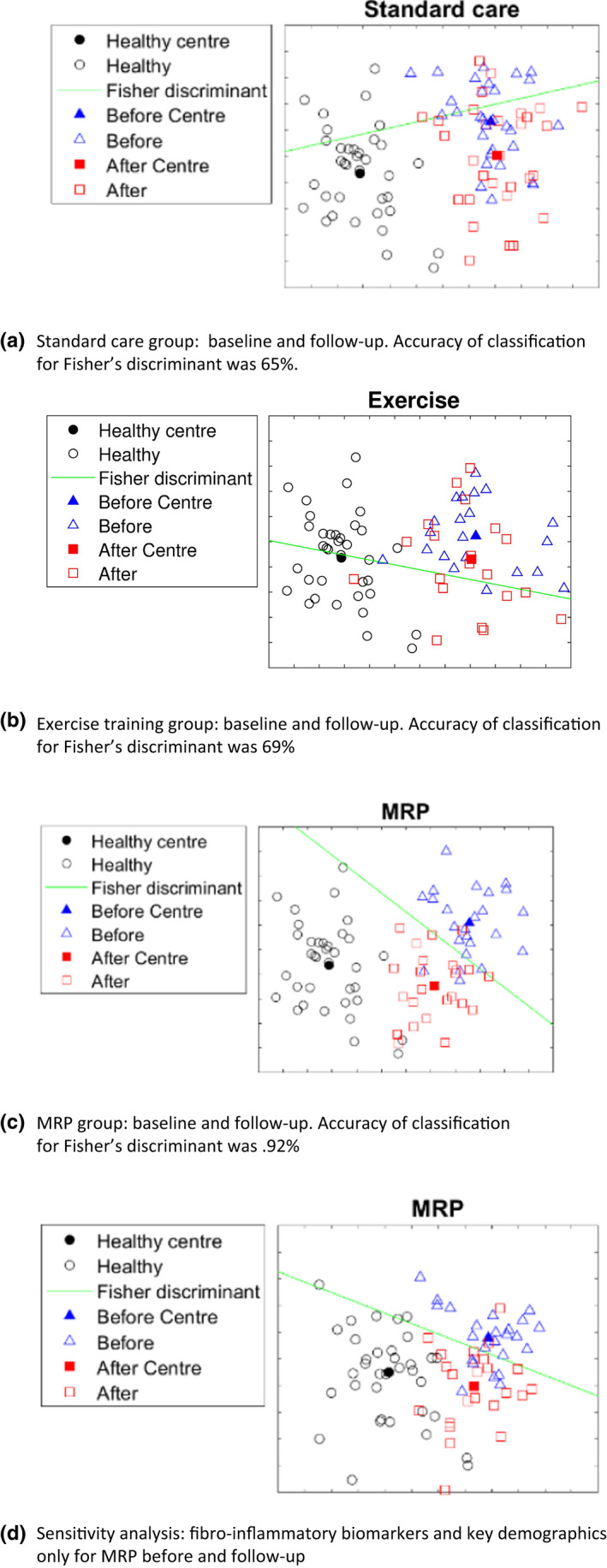
(a–d) Fishers Linear Discriminant and 2D‐visulisation for each group pre and post‐intervention. (a) Standard care group: baseline and follow‐up. Accuracy of classification for Fisher's discriminant was 65%. (b) Exercise training group: baseline and follow‐up. Accuracy of classification for Fisher's discriminant was 69%. (c) MRP group: baseline and follow‐up. Accuracy of classification for Fisher's discriminant was 0.92%. (d) Sensitivity analysis: fibro‐inflammatory biomarkers and key demographics only for MRP before and follow‐up.

### 3.4 Pre‐ and post‐intervention

A total of 90 participants with T2D were enrolled into the RCT of which 76 went on to complete the trial—30 received standard care, 24 the MRP and 22 exercise training (Figure 1). The sub‐groups were well balanced at baseline as expected (Figure S4). The main outcomes of the DIASTOLIC study have been reported^7^ and provided in Table S6. Briefly, the MRP group saw significant cardio‐metabolic improvements including weight loss (13.6 Kg), blood pressure (13 mmHg systolic), arterial stiffness, reduced concentric remodelling, insulin resistance, fasting glucose (−1.9 mmol), with 20 (83%) participants in this group achieving T2D remission by 12 weeks. Remission was defined as a fasting glucose of <7.0 mmol/L or HbA1c < 6.5% without requirement for hypoglycaemic agents post‐intervention.^12^ In the exercise training arm there was a small reduction in weight (1.6 Kg) and fasting glucose (−0.8 mmol/L) and a significant improvement in diastolic function.^7^

This differential response to the MRP and exercise training intervention extended to the plasma biomarkers. In the MRP group six biomarkers associated with inflammation (leptin, adiponectin, FABP4, NGAL, OPN, PTX‐3) five with fibrosis (MMP2, 8 and 9, CHI3L1, PAI1), three with endothelial function (endostatin, ET1, VEGFR1) and one associated with atrial stretch (NTpro‐ANP) were altered at 12 weeks, whether or not adjusted for change in weight or HbA1c (Table S7). However, only two plasma biomarkers associated with fibrosis (MMP8 and 9) were significantly changed in the exercise training group at 12 weeks, again with or without these adjustments (Table S7).

### 3.5 Pre‐ and post‐visualisation [Figure 3(a‐d)]

The only group where Fisher's discriminant produced reasonable separation pre and post‐intervention was in the MRP group (Figure 2c). Furthermore, in this visualisation the post‐MRP cluster shifts closer to the healthy controls. Thirteen variables were present in both axes for change including: five biomarkers, three MRI measures, and three measures of glycaemic control, weight and BMI (Table S8a–b). To account for this separation and shift towards healthy profile not being driven only by changes in weight or glycaemic status, the visualisation was repeated with fibro‐inflammatory biomarkers and key demographics only. The resultant fisher's discriminant analysis again produced a separation which is attenuated but remains evident (Figure 2d). The accuracy of classification for Fisher's discriminant for standard care was 65%, for exercise training 69% and for MRP 92%.

## 4 DISCUSSION

This post hoc analysis of DIASTOLIC shows working age people with a relatively short duration of T2D without CVD having a different fibro‐inflammatory and cardiovascular profile to metabolically healthy controls. Those with T2D also demonstrated evidence of diastolic dysfunction and concentric remodelling indicative of early diabetic heart failure. Following a 12 week lifestyle intervention those in the supervised exercise training programme demonstrated an improvement in diastolic function in the absence of any significant weight‐loss, normalisation of glycaemic control, or changes in fibro‐inflammatory profiles. Contrastingly, the MRP treatment led to significant weight‐loss coupled with reversal of T2D, in the majority of cases, with evidence of early improvements in concentric remodelling/aortic stiffness and significant changes to their fibro‐inflammatory status; although the biomarkers did not fully reach the levels seen in healthy controls within the relatively short treatment period.

The data we report here adds further evidence for the potential mechanistic role of fibrosis and inflammatory processes in the pathophysiology of diabetic cardiomyopathy, suggesting that early intervention to ameliorate glycometabolic derangements in T2D may reverse this fibro‐inflammatory profile.

### 4.1 Fibro‐inflammatory biomarker profiles case–control

A different relationship between the fibro‐inflammatory biomarkers and MRI measures exists in those with T2D compared to healthy controls, highlighted by the almost complete separation based on the principal component reduction. There is evidence of cardiac remodelling in those with T2D, who overall had an adverse fibro‐inflammatory profile compared to healthy controls. The upregulation of the interstitial fibrotic markers MMP‐7, MMP‐8 and plasminogen activator inhibitor‐1 (PAI1) observed in people with T2D is indicative of a pro‐fibrotic environment. MMPs −7 and − 8 play critical roles in the maintenance of the extracellular matrix (ECM) through degradation and turnover of collagens, regulation of cell migration and tissue repair. Elevated MMP‐7 levels have been reported in patients with T2D without CVD, with evidence of diastolic dysfunction.^13^ PAI‐1 increases in response to a pro‐inflammatory state,^14^ as recognised in people with T2D,^15^ with significantly higher levels previously reported with incident CVD vs. without.^16^

The down‐regulation of MMP‐2 in those with T2D could be attributed to their elevated HbA1c levels.^17^ Likewise, lower MMP‐3 levels have been reported in obese individuals albeit without T2D.^18^ However the large difference in mean values of BMI between cases and controls in our study could explain the observed difference. The lower levels of Tenascin C in those with T2D is surprising. Tenascin C is a pro‐inflammatory and fibrotic ECM glycoprotein secreted by endothelial cells including those of the aorta and pulmonary artery.^19^ Recently, elevated serum levels have been reported in adults with T2D.^20^ We speculate that Tenascin C levels may not drive development of fibrosis in early diabetic cardiomyopathy and may not be sensitive markers for identifying alterations at this stage of disease.

It is known that T2D is a state of subclinical inflammation with specific reductions in adiponectin and other markers associated with endothelial dysfunction.^21^, ^22^ The observed lower levels of acute phase inflammatory glycoprotein PTX‐3 in the T2D group is unexpected, especially given the raised levels of TNF‐alpha, an inducer of PTX‐3.^23^ There is evidence supporting a role for PTX‐3 in the development of CVD,^23^ but also evidence of the induction of PTX‐3 by anti‐inflammatory and atheroprotective signals, which would be consistent with our findings. Knock‐out (PTX‐3) mouse models have shown vascular wall inflammation compared to those with PTX‐3 expression.^24^ One must consider the lower levels resulting from the glucose lowering medication these patients were taking. Metformin, the first line therapy for T2D, is increasingly being recognised as an anti‐inflammatory agent.^25^, ^26^

N‐terminal pro‐atrial natriuretic peptide (NTproANP) is the precursor of atrial natriuretic peptide and is secreted in response to atrial expansion due to increased blood volume and/or increased intra‐atrial pressure. The left atrial maximal volumes in the T2D group were significantly lower than the healthy controls, consistent with the lower levels observed of this biomarker, despite LV filling pressures being higher, as we have also observed in HFpEF.^5^

### 4.2 Differential fibro‐inflammatory response to exercise training and dietary intervention

Our data show a differential effect of the MRP and exercise training interventions in people with T2D and early asymptomatic CVD on fibro‐inflammatory biomarkers. There were markedly more alterations in these biomarkers following the MRP compared to the exercise training intervention, even after adjustment for changes in weight and glycaemic control. Whilst there was an improvement in diastolic function, which was the primary outcome measure of the trial, following the supervised exercise training programme no significant weight‐loss or normalisation of glycaemic control was observed that could, in part, be attributed to the differences in energy expenditure between the groups. Further, the visualisations of Fisher's discriminant indicate that the relationship between MRI outcomes, body composition, glycaemia and fibro‐inflammatory biomarkers did not change. Comparatively, a distinct change in these relationships was observed following the MRP, as demonstrated by the separation of the two clusters. The MRP was associated with a significant weight‐loss, improved glycaemic control, blood pressure and markers of diabetic cardiomyopathy (arterial stiffness and concentric remodelling). Finally, the data indicate a shift towards the healthy phenotype following the 12 weeks intervention in the MRP group only.

The data presented are suggestive of additional benefits of the MRP from those previously reported^7^ related to the inflammatory state of T2D. Interestingly there was not a complete normalisation of these biomarkers perhaps attributed to insufficient follow‐up time. Indeed it is increasingly recognised that rapid weight‐loss through low‐energy diets, such as with this MRP, induces an initial negative impact on cardiac performance thought to be driven by an influx of free fatty acids (FFAs) to the myocardium. This elicits a shift in cardiac energetics that normalises over time followed by improved cardiac function.^27^ Therefore, the longer‐term benefits of a successful MRP intervention in people with T2D could lead to reduced cardiovascular outcomes through alterations in fibro‐inflammatory pathways and reversal of markers of diabetic cardiomyopathy. Longer term studies with larger cohorts are required to further explore and confirm this. Maintenance and/or rescue strategies should be incorporated in to longer term trials to ensure weight‐loss is sustainable beyond the 12 week MRP programme. Further, the impact of combining MRP with an exercise training program should be explored in future studies to determine if additional benefits arise compared to the MRP alone. The impact of glucose lowering therapies on the circulating levels of these biomarkers also warrants further exploration in longer term studies.

### 4.3 Strengths and limitations

The major strength and novelty of this study lies in the comprehensive phenotyping of the cohort with MRI (the gold standard technique for assessment of cardiovascular structure and function) and quantification of 51 fibro‐inflammatory biomarkers utilising data reduction techniques with 2 and 3D‐visualisations accompanied by methodical between and within group comparisons. The DIASTOLIC study was a powered RCT with well‐balanced group allocation. Limitations relate to the exploratory nature of the study given the main trial was not powered to detect differences in plasma biomarkers between groups, therefore some signals may be blunted. Further, given this is plasma analysis, we cannot determine where each biomarker is secreted, in response to which stimuli nor what downstream signalling it elicits. The MRP had a greater calorie deficit than the exercise training group which could also be a contributory factor to the observed differences at 12 weeks between the groups. The nature of data reduction techniques requires a trade‐off between accuracy and simplicity. However, these are well‐established mathematical methods that permit the exploration and visualisation of large complex datasets. The relatively short duration of the interventions limits our ability to assess longer‐term benefits, although an MRP has been shown to confer sustained weight loss and improved glycaemic control at 2 years.^28^

In conclusion, the fibro‐inflammatory and cardiovascular profile of people with T2D free of CVD is different to healthy controls. A 12‐week MRP in T2D results in a dramatic shift in the relationship between fibro‐inflammatory markers and subtle markers of diabetic heart failure towards the healthy profile. These results suggest potential for improvement in cardiovascular outcomes which should be ascertained in larger studies with long‐term follow‐up.

## AUTHOR CONTRIBUTIONS

See main outcomes paper for contributions to the original study. For these analysis and the manuscript, all authors contributed to the development of the research questions addressed. JY and LZ undertook the quantification of the plasma biomarkers. EMB, EMM and LG analysed the data. EMB drafted the manuscript with input from MPMGB, EMM, GSG, PK, LN and GPM. All authors critically revised the manuscript and contributed to subsequent revisions. All authors approved the final version.

## GUARANTOR STATEMENT

**EMB, EMM, GPM** are the guarantors of this work and, as such, had full access to all the data in the study and take responsibility for the integrity of the data and the accuracy of the data analysis.

## FUNDING INFORMATION

This study was funded by the NIHR Research Trainees Coordinating Centre through a career development fellowship to GPM (CDF 2014‐07‐045) and supported by the NIHR Leicester Biomedical Research Centre and Clinical Research Facility. BSM subsidised the cost of the biomarker analysis.

## CONFLICT OF INTEREST

Cambridge Weight Plan provided the dietary supplements free of charge but was not involved in the conduct of the study, analysis or interpretation of the data, or writing of the report. JY and LZ are employed by Bristol Myers Squibb (BSM). No other potential conflicts of interest relevant to this article were reported.

## PRIOR PRESENTATION

Fibro‐inflammatory plasma biomarkers and relation to cardiovascular remodelling in type 2 diabetes (June 2021). Presented at the American Diabetes Association 81st Scientific Sessions: oral abstract session entitled Pathogenesis of Atherosclerosis and Its Complications in Diabetes (With Edwin Bierman Award Lecture, 56‐OR)).

## Supporting information


Appendix S1
