# Supplementary material for: Fibro‐inflammatory recovery and type 2 diabetes remission following a low calorie diet but not exercise training: A secondary analysis of the DIASTOLIC randomised controlled trial
Source: Diabet Med. 2022 Jun 16;39(8):e14884. doi: 10.1111/dme.14884 (PMC9543965; doi:10.1111/dme.14884)
Supplement: Supplementary file 1 — Appendix S1 [file DME-39-0-s001.docx]

# Supplementary material

## DIASTOLIC inclusion/exclusion criteria

Inclusion Criteria

1. Capacity to provide informed consent
2. Established T2D (≥3months)
3. Age ≥18and ≤65years
4. HbA1c >6*.*5% and ≤9% if on triple therapy or ≤10% on diet and exercise or monotherapy or dual therapy
5. Current glucose-lowering therapy either mono, dual or triple of any combination of metformin, sulphonylurea, DPP-IV inhibitor, GLP-1 therapy or an SGLT2i ± diet and exercise
6. BMI >30kg/m2 or >27kg/m2 (South Asian)
7. Diagnosis of T2D before the age of 60years of age

Exclusion Criteria

1. Diabetes duration >12years
2. Currently taking more than three glucose-lowering therapies
3. Weight loss of >5kg in the preceding 6months
4. Stage 4 or 5 chronic kidney disease (eGFR <30mL/min/1*.*73m^2^)
5. Current therapy with insulin, thiazolidinediones, steroids or atypical antipsychotic medication
6. Untreated thyroid disease
7. Known macrovascular disease including coronary artery disease, stroke/TIA or peripheral vascular disease
8. Presence of arrhythmia (including atrial fibrillation, atrial flutter or second or third degree atrioventricular block)
9. Known heart failure or other clinically relevant heart disease
10. Inability to exercise or undertake a MRP
11. Absolute contraindication to CMRI
12. Cardiovascular symptoms (angina and limiting dyspnoea during normal physical activity)
13. Inflammatory condition, for example, connective tissue disorder and rheumatoid arthritis.

## DIASTOLIC interventions

Random allocation (1:1:1) was stratified by sex and glucose lowering therapy by an independent online computerized randomization system incorporating concealed allocation (Sealed Envelope).

Interventions

The exercise group: Participants attended 50 minute supervised sessions thrice weekly at the Leicester Biomedical Research Centre. Each exercise session consisted of a warm-up, stimulus (walking or cycling) and cool-down phase. The duration of exercise in each training mode was increased gradually to achieve the 50 min target. Cardiorespiratory fitness was assessed at baseline and exercise intensity titrated using the value from the baseline peak VO2 assessment to achieve ~60% VO2max at each session. Objective (heart rate monitoring) and subjective (Borg Rate of Perceived Exertion Scale, graded from 6 to 20) measures were used to measure responses to the exercise sessions with individual exercise intensity adjusted progressively throughout the 12 weeks to take account of increasing fitness levels. Compliance was considered as ≥ two-thirds attendance. A medication review was performed by an appropriately trained clinician and alterations made where clinically indicated.

MRP group: Participants received a nutritionally balanced low energy diet of ~810 kcal/day (30% protein, 50% carbohydrate, 20% fat) (Cambridge Weight Plan) with alterations to their glucose lowering and anti-hypertensive therapies as described in the protocol paper.1 All MRP participants received health behaviour coaching with relapse prevention via weekly contact. The diet was stopped, and a maintenance diet introduced once 50% excess body weight was achieved, or by 12 weeks, whichever came first. Compliance was considered if appropriate weight-loss was achieved at pre-defined time points (>2% body weight at week 1 and 4% at week 3). For either intervention any non-compliant participants were withdrawn.

Control group: Participants received lifestyle advice in a single health coaching interview at baseline and 12 weeks. Advice was in accordance with the National Institute for Health and Care Excellence guidance.^2^

A summary of baseline medication is listed below.

| **Medication** | **Number of participants receiving therapy (n=83)** | **Treatment regimen for diabetes** | |
| --- | --- | --- | --- |
| **Glucose Lowering** | | | |
| Metformin | 78 | Diet and lifestyle only | 3 |
| Sulphonylurea | 12 | Monotherapy | 44 |
| Gliptin | 9 | Dual therapy | 26 |
| GLP-1 | 17 | Triple therapy | 10 |
| SGLT2 inhibitor | 10 |  | |
| Insulin | 0 |  |  |
| **Anti-hypertensives** | |  |  |
| Ace inhibitor | 27 |  |  |
| Alpha blocker | 3 |  |  |
| ARB | 11 |  |  |
| Beta-blocker | 6 |  |  |
| Calcium channel blocker | 19 |  |  |
| **Cholesterol lowering** |  |  |  |
| Statin | 55 |  |  |
| Fibrate | 3 |  |  |

Abbreviations: GLP-1 = Glucagon-like peptide -1, SGLT2 = sodium-glucose cotransporter-2, ARB= Angiotensin receptor blocker.

## Plasma biomarkers of interest

The following plasma biomarkers implicated in heart failure (HF) were quantified using multiplex array Luminex® xMAP technology. ^3^ Angiopoietin, pro-BNP, highly sensitive C-reactive protein (CRP), chitinase 3-like 1 (CHI3L1), cystatin-C, endoglin, endostatin, endothelin-1 (ET-1), Fatty-acid-binding proteins (FABP3 and 4 (heart and adipocyte types respectively)), Fibroblast growth factors (FGF21 and 23), Fas, growth differentiation factor-15 (GDF-15), galectin 3, intercellular adhesion molecule-1 (ICAM-1), interleukins ( IL1 beta, IL10, IL6 and IL8), matrix metalloproteinases (MMP-2, MMP-3, MMP-7, MMP-8, MMP-9, MMP-12), tissue inhibitor of metalloproteinases (TIMP-1, TIMP-4), Neutrophil gelatinase associated lipocalin (NGAL), myeloperoxidase (MPO), N-terminal pro-atrial natriuretic peptide (NTpro-ANP), N-terminal pro-brain natriuretic peptide (NTpro-ANP), osteoprotegerin (OPG), osteopontin (OPN), p-selectin, Plasminogen activator inhibitor-1 (PAI-1), pentraxin-3 (PTX3), renin, suppression of tumorigencity-2 (ST2), syndecans (1 and 4), Kidney Injury Molecule-1 (KIM-1), tumour necrosis factor receptors (TNFR1 and 2), tumour necrosis factor alpha (TNF-a), tenascin C, troponin T, and Vascular endothelial growth factor alpha (VEGFa).

All plasma biomarkers (total 51 to include adiponectin and leptin) were then further categorized as either surrogates of myocardial interstitial fibrosis, Left Ventricular (LV) cardiomyocyte stress/damage, myocardial hypertrophy, inflammation/oxidative stress, atrial wall stress/stretch or markers of renal dysfunction.

## Cardiac MRI outcome measures

The following CMR outcomes of interest were analysed: LV End Diastolic Volume (LV EDV (ml)), LV mass (LVM, g), LV mass to volume ratio (LV mass/volume (g/mL), LV Global Longitudinal Strain ( LV GLS (%)), LV Global Circumferential Strain (LV GCS (%)), LV Longitudinal Peak Early Diastolic Strain rate (LV LongPEDSR (s-1)), LV Circumferential Peak Early Diastolic Strain rate (LV CircPEDSR (s-1)), Left Atrial maximal volume (LAmax V (mL/m2)), LA Ejection Fraction (LA EF (%)), LV Ejection Fraction (LV EF (%)), mean aortic distensibility (mmHg-1 x 10-3), myocardial perfusion reserve (MPR (calculated using Fermi-constrained deconvolution^4^)) and presence of Late Gadolinium Enhancement (LGE (assessed qualitatively as absent or present) to detect focal myocardial fibrosis.

## Supplementary tables 1- 6

Supplementary Table 1. Correlation of CMR outcomes of interest and each biomarker for participants with T2D

| Biomarker | LV EDVi | LV massi | LV m:v | LV GLS | LV long PEDSR | LV circ PEDSR | LV GCS | LAVImax | LA EF | E/e' | LGE | Global MPR | Mean AD |
| --- | --- | --- | --- | --- | --- | --- | --- | --- | --- | --- | --- | --- | --- |
| Adiponectin | -0*.*18 | -0*.*13 | 0*.*02 | -0*.*05 | 0*.*03 | -0*.*09 | -0*.*01 | 0*.*08 | -0*.*07 | 0*.*18 | -0*.*05 | -0*.*17 | 0*.*04 |
| Angiopoietin | 0*.*19 | 0*.*18 | 0*.*00 | 0*.*03 | -0*.*09 | -0*.*02 | 0*.*18 | 0*.*11 | -0*.*13 | -0*.*01 | -0*.*05 | 0*.*00 | 0*.*03 |
| BNP | -0*.*02 | 0*.*02 | 0*.*06 | 0*.*13 | -0*.*02 | 0*.*07 | 0*.*11 | 0*.*03 | -0*.*08 | -0*.*16 | 0*.*01 | 0*.*00 | 0*.*07 |
| CRP | -0*.*17 | -0*.*12 | 0*.*02 | 0*.*18 | -0*.*08 | 0*.*12 | -0*.*03 | 0*.*00 | -0*.*13 | 0*.*06 | 0*.*08 | -0*.*23 | -0*.*04 |
| CHI3L | -0*.*03 | 0*.*08 | 0*.*13 | 0*.*23 | -0*.*08 | -0*.*17 | 0*.*00 | -0*.*14 | -0*.*04 | -0*.*05 | 0*.*14 | 0*.*05 | -0*.*26 |
| Cystatin C | -0*.*14 | 0*.*03 | 0*.*17 | 0*.*09 | 0*.*00 | -0*.*17 | 0*.*06 | -0*.*06 | -0*.*10 | 0*.*21 | -0*.*02 | -0*.*17 | -0*.*12 |
| Endoglin | 0*.*09 | 0*.*18 | 0*.*12 | 0*.*16 | -0*.*10 | -0*.*09 | 0*.*16 | -0*.*01 | -0*.*15 | -0*.*21 | 0*.*12 | 0*.*31 | 0*.*06 |
| Endostatin | -0*.*11 | -0*.*08 | 0*.*03 | 0*.*00 | -0*.*02 | -0*.*06 | -0*.*04 | 0*.*04 | -0*.*21 | 0*.*13 | -0*.*14 | -0*.*20 | 0*.*01 |
| ET1 | -0*.*06 | -0*.*13 | -0*.*06 | -0*.*15 | 0*.*20 | 0*.*16 | -0*.*18 | -0*.*11 | 0*.*05 | 0*.*04 | 0*.*03 | 0*.*09 | 0*.*05 |
| FABP3 | 0*.*06 | -0*.*02 | -0*.*07 | 0*.*05 | -0*.*12 | 0*.*11 | 0*.*04 | 0*.*19 | -0*.*13 | 0*.*04 | -0*.*02 | -0*.*06 | -0*.*01 |
| FABP4 | -0*.*17 | -0*.*24 | -0*.*10 | -0*.*08 | 0*.*10 | 0*.*26 | -0*.*21 | -0*.*13 | 0*.*03 | 0*.*14 | -0*.*11 | -0*.*18 | 0*.*04 |
| FGF21 | 0*.*16 | 0*.*09 | -0*.*06 | 0*.*09 | 0*.*00 | 0*.*05 | 0*.*08 | -0*.*02 | 0*.*07 | -0*.*09 | 0*.*24 | 0*.*06 | 0*.*01 |
| FGF23 | -0*.*12 | 0*.*00 | 0*.*11 | 0*.*06 | -0*.*12 | -0*.*01 | -0*.*04 | 0*.*00 | -0*.*09 | 0*.*05 | -0*.*24 | -0*.*17 | -0*.*07 |
| Fas | -0*.*11 | -0*.*08 | 0*.*03 | -0*.*03 | -0*.*08 | -0*.*15 | 0*.*04 | -0*.*08 | -0*.*03 | -0*.*06 | 0*.*04 | 0*.*12 | -0*.*12 |
| GDF15 | -0.04 | 0.08 | 0.12 | 0.17 | -0.03 | -0.08 | 0.12 | -0.07 | -0.08 | 0.05 | 0.05 | 0.16 | -0.25 |
| Galectin 3 | -0.28 | -0.31 | -0.06 | -0.06 | -0.11 | 0.06 | 0.01 | -0.01 | 0.01 | 0.06 | -0.27 | -0.12 | 0.12 |
| ICAM1 | 0.14 | 0.13 | 0.00 | -0.06 | 0.11 | 0.05 | 0.05 | -0.11 | 0.00 | 0.13 | 0.14 | 0.07 | 0.00 |
| IL1 beta | 0.09 | 0.14 | 0.03 | -0.20 | 0.04 | 0.04 | -0.15 | 0.08 | -0.09 | -0.04 | -0.01 | 0.05 | 0.01 |
| IL10 | 0.20 | 0.20 | 0.03 | 0.01 | -0.04 | -0.08 | -0.07 | -0.04 | -0.08 | -0.03 | -0.10 | 0.07 | -0.09 |
| IL6 | -0.11 | -0.09 | 0.02 | -0.04 | 0.03 | 0.07 | -0.13 | -0.17 | -0.03 | 0.18 | -0.12 | -0.18 | 0.00 |
| IL8 | -0.15 | -0.09 | 0.08 | 0.19 | -0.19 | -0.11 | 0.15 | -0.17 | -0.05 | -0.06 | 0.09 | 0.21 | -0.09 |
| NGAL | 0.14 | -0.06 | -0.21 | 0.02 | -0.16 | -0.10 | 0.04 | 0.00 | -0.12 | -0.04 | -0.15 | -0.14 | 0.04 |
| MMP12 | -0.03 | -0.03 | 0.00 | 0.01 | -0.02 | 0.01 | 0.01 | -0.01 | -0.10 | -0.04 | 0.11 | 0.07 | 0.00 |
| MMP2 | -0.11 | -0.05 | 0.04 | 0.03 | -0.10 | -0.08 | 0.10 | 0.07 | -0.08 | -0.04 | -0.02 | -0.03 | -0.02 |
| MMP3 | 0.00 | 0.06 | 0.07 | 0.04 | -0.10 | -0.12 | 0.07 | -0.11 | 0.02 | -0.12 | 0.02 | -0.01 | 0.00 |
| MMP7 | -0.29 | -0.07 | 0.22 | 0.12 | -0.06 | -0.02 | -0.02 | -0.07 | -0.10 | 0.17 | -0.13 | 0.00 | -0.26 |
| MMP8 | 0.05 | -0.01 | -0.04 | 0.14 | -0.04 | 0.14 | 0.04 | -0.20 | 0.01 | -0.26 | 0.11 | 0.12 | 0.09 |
| MMP9 | -0.03 | -0.18 | -0.17 | -0.05 | 0.00 | 0.07 | 0.11 | -0.16 | 0.04 | -0.12 | 0.00 | 0.08 | 0.10 |
| MPO | -0.08 | -0.03 | 0.03 | 0.06 | -0.01 | -0.01 | 0.10 | -0.05 | -0.10 | 0.15 | -0.09 | -0.13 | -0.02 |
| NTproANP | -0.02 | 0.09 | 0.11 | -0.04 | -0.03 | -0.04 | -0.09 | 0.19 | -0.06 | 0.04 | -0.25 | 0.25 | -0.05 |
| NTproBNP | 0.00 | 0.25 | 0.29 | 0.14 | -0.26 | -0.12 | 0.05 | 0.12 | -0.15 | 0.06 | 0.00 | 0.01 | 0.01 |
| OPG | -0.05 | 0.13 | 0.19 | 0.11 | -0.05 | -0.11 | 0.12 | 0.05 | 0.01 | -0.22 | 0.01 | 0.13 | -0.26 |
| OPN | 0.07 | 0.13 | 0.04 | 0.00 | -0.04 | -0.04 | 0.06 | 0.22 | -0.01 | -0.02 | -0.05 | 0.21 | -0.04 |
| p Selectin | -0.21 | -0.25 | -0.04 | -0.01 | 0.00 | 0.14 | -0.11 | -0.04 | 0.09 | -0.02 | 0.08 | -0.06 | 0.04 |
| PAI1 | -0.08 | 0.00 | 0.06 | 0.08 | 0.08 | -0.03 | -0.10 | -0.09 | 0.03 | -0.05 | 0.05 | -0.01 | -0.26 |
| Pentraxin 3 | 0.10 | 0.07 | 0.01 | 0.10 | -0.04 | -0.06 | 0.05 | -0.16 | -0.18 | -0.25 | 0.22 | -0.02 | 0.09 |
| Renin | 0.07 | 0.04 | -0.01 | 0.11 | -0.15 | -0.16 | 0.09 | -0.18 | -0.08 | -0.19 | 0.17 | 0.15 | 0.06 |
| ST2 | 0.01 | 0.13 | 0.16 | 0.23 | -0.14 | -0.05 | 0.09 | -0.12 | 0.05 | -0.05 | 0.07 | 0.33 | -0.16 |
| Syndecan 1 | 0.15 | 0.03 | -0.14 | -0.01 | -0.01 | 0.05 | 0.01 | 0.25 | -0.06 | -0.05 | -0.14 | -0.08 | 0.03 |
| Syndecan 4 | 0.06 | -0.02 | -0.11 | -0.05 | 0.11 | 0.09 | -0.01 | 0.16 | 0.07 | 0.02 | -0.15 | -0.02 | -0.06 |
| KIM1 | -0.03 | -0.09 | -0.06 | 0.09 | 0.10 | 0.11 | 0.11 | 0.01 | -0.19 | -0.14 | -0.09 | -0.10 | -0.04 |
| TIMP1 | 0.00 | -0.02 | -0.05 | 0.05 | 0.04 | -0.11 | 0.00 | 0.06 | -0.06 | 0.11 | -0.02 | -0.12 | -0.06 |
| TIMP4 | -0.10 | -0.03 | 0.07 | -0.02 | 0.23 | 0.04 | -0.12 | 0.01 | -0.14 | 0.25 | -0.12 | -0.25 | -0.06 |
| TNFR1 | -0.16 | -0.15 | 0.01 | 0.06 | -0.02 | 0.03 | -0.13 | -0.15 | -0.09 | 0.29 | -0.19 | -0.15 | -0.16 |
| TNFR2 | -0.11 | 0.01 | 0.12 | 0.07 | -0.08 | -0.15 | 0.08 | -0.08 | -0.07 | 0.24 | 0.01 | -0.11 | -0.12 |
| TNF alpha | -0.05 | -0.06 | 0.00 | 0.11 | -0.18 | 0.04 | -0.05 | -0.07 | -0.08 | -0.04 | -0.07 | 0.09 | 0.02 |
| Tenascin C | -0.20 | -0.15 | 0.04 | 0.08 | -0.09 | -0.02 | 0.06 | 0.03 | 0.00 | -0.07 | -0.11 | -0.11 | 0.07 |
| Troponin T | 0.09 | 0.11 | 0.02 | -0.14 | 0.06 | 0.17 | -0.22 | 0.09 | 0.15 | 0.00 | -0.05 | 0.17 | -0.03 |
| VEGFR1 | 0.01 | 0.09 | 0.08 | 0.01 | -0.07 | -0.07 | 0.03 | 0.12 | 0.04 | -0.26 | -0.11 | 0.22 | -0.05 |
| VEGFa | 0.05 | 0.10 | 0.06 | -0.03 | 0.08 | 0.03 | -0.09 | -0.08 | 0.07 | 0.13 | 0.02 | 0.12 | -0.20 |
| Leptin | -0.29 | -0.29 | -0.03 | -0.15 | 0.09 | 0.16 | -0.20 | 0.03 | -0.07 | 0.24 | -0.11 | -0.28 | -0.01 |

Data reported as Pearson’s correlation co-efficient (r) green background highlighted *p*-value less than ≤0.01, yellow background highlighted *p*-value less than ≤0.05

Supplementary Table 2. Correlation of CMR outcomes of interest and each biomarker for healthy participants

| Biomarker | LV EDVi | LV massi | LV m:v | LV GLS | LV long PEDSR | LV circ PEDSR | LV GCS | LAVImax | LA EF | E/e' | LGE | Global MPR | Mean AD |
| --- | --- | --- | --- | --- | --- | --- | --- | --- | --- | --- | --- | --- | --- |
| Adiponectin | -0.13 | -0.20 | -0.17 | -0.02 | 0.23 | -0.16 | -0.16 | 0.09 | 0.07 | 0.24 | -0.28 | -0.17 | 0.26 |
| Angiopoietin | 0.05 | 0.06 | -0.02 | 0.12 | -0.26 | -0.01 | -0.20 | 0.20 | -0.00 | 0.18 | 0.20 | 0.13 | 0.09 |
| BNP | -0.02 | 0.02 | 0.06 | -0.08 | 0.07 | 0.03 | 0.20 | 0.04 | -0.01 | 0.13 | 0.05 | 0.12 | -0.03 |
| CRP | 0.02 | -0.02 | -0.01 | -0.03 | 0.16 | 0.15 | 0.14 | 0.08 | 0.17 | -0.20 | -0.07 | -0.06 | 0.11 |
| CHI3L | 0.27 | 0.10 | -0.30 | -0.01 | -0.18 | -0.07 | -0.16 | 0.20 | -0.29 | -0.16 | -0.17 | 0.12 | 0.10 |
| Cystatin C | 0.11 | 0.02 | -0.12 | 0.24 | -0.18 | -0.32 | 0.14 | -0.12 | -0.05 | -0.05 | -0.08 | 0 | 0.34 |
| Endoglin | 0.25 | 0.27 | 0.12 | 0.18 | -0.21 | 0.04 | 0.01 | 0.22 | 0.05 | 0.18 | 0.15 | 0.07 | 0.14 |
| Endostatin | -0.10 | -0.10 | -0.01 | -0.01 | -0.11 | 0.07 | -0.21 | 0.14 | -0.12 | 0.12 | 0.10 | 0.14 | -0.23 |
| ET1 | -0.08 | -0.01 | 0.15 | 0.12 | -0.05 | 0.13 | -0.25 | -0.04 | -0.11 | 0.12 | 0.02 | -0.09 | -0.21 |
| FABP3 | 0.01 | 0.13 | 0.25 | -0.09 | -0.12 | 0.26 | -0.04 | 0.02 | -0.03 | 0.13 | 0.14 | 0.03 | -0.24 |
| FABP4 | -0.10 | -0.23 | -0.25 | -0.25 | 0.30 | 0.14 | -0.24 | -0.02 | 0.01 | 0.36 | 0.06 | 0.07 | -0.04 |
| FGF21 | 0.02 | 0.03 | 0.10 | 0.01 | 0.09 | 0.11 | 0.16 | -0.03 | -0.16 | 0.05 | -0.11 | 0.1 | 0.05 |
| FGF23 | -0.15 | -0.17 | -0.09 | -0.11 | 0.00 | 0.07 | -0.30 | -0.05 | -0.02 | 0.31 | 0.02 | -0.01 | 0.07 |
| Fas | 0.11 | 0.08 | -0.01 | 0.09 | -0.22 | -0.04 | -0.12 | -0.01 | 0.11 | 0.08 | -0.25 | 0.18 | -0.03 |
| GDF15 | -0.23 | -0.21 | 0.04 | -0.06 | -0.06 | -0.03 | -0.28 | 0.26 | 0.16 | 0.33 | -0.10 | -0.04 | -0.20 |
| Galectin 3 | -0.13 | -0.13 | -0.02 | -0.20 | 0.14 | 0.12 | -0.23 | -0.16 | 0.04 | 0.15 | -0.01 | 0.19 | -0.05 |
| ICAM1 | -0.19 | -0.13 | 0.07 | -0.25 | 0.06 | 0.19 | -0.38 | -0.13 | 0.06 | 0.23 | 0.35 | 0 | -0.08 |
| IL1 beta | -0.06 | 0.01 | 0.13 | -0.12 | 0.27 | 0.31 | -0.23 | -0.18 | 0.43 | -0.01 | -0.04 | -0.08 | 0.05 |
| IL10 | -0.06 | -0.04 | 0.01 | 0.04 | -0.01 | 0.21 | 0.08 | -0.02 | 0.11 | -0.10 | 0.30 | -0.04 | -0.09 |
| IL6 | 0.14 | 0.18 | 0.14 | 0.20 | -0.14 | 0.09 | 0.21 | 0.19 | -0.12 | -0.12 | 0.07 | -0.03 | -0.20 |
| IL8 | -0.22 | -0.20 | 0.04 | -0.09 | 0.15 | 0.23 | -0.13 | -0.11 | 0.02 | -0.08 | 0.38 | 0.04 | -0.23 |
| NGAL | 0.09 | 0.03 | -0.13 | 0.09 | -0.30 | -0.16 | -0.14 | -0.08 | -0.10 | -0.00 | -0.05 | 0.32 | 0.01 |
| MMP12 | -0.09 | -0.16 | -0.13 | 0.26 | 0.00 | 0.07 | -0.21 | -0.06 | -0.12 | 0.09 | 0.07 | -0.2 | -0.17 |
| MMP2 | 0.14 | 0.03 | -0.18 | 0.07 | -0.06 | 0.13 | -0.24 | 0.23 | 0.15 | 0.18 | -0.23 | 0.07 | 0.17 |
| MMP3 | 0.10 | 0.19 | 0.13 | 0.17 | -0.16 | 0.01 | 0.26 | -0.25 | 0.06 | -0.30 | 0.08 | 0.33 | 0.12 |
| MMP7 | 0.06 | 0.10 | 0.14 | 0.22 | -0.11 | -0.04 | 0.07 | -0.01 | -0.09 | 0.01 | -0.00 | -0.01 | -0.04 |
| MMP8 | 0.13 | 0.15 | 0.14 | 0.08 | -0.21 | -0.11 | -0.06 | 0.13 | -0.10 | 0.03 | 0.26 | 0.13 | -0.20 |
| MMP9 | -0.15 | -0.18 | -0.02 | -0.19 | 0.00 | 0.07 | -0.39 | -0.01 | -0.07 | 0.09 | 0.08 | 0.13 | -0.01 |
| MPO | -0.00 | -0.09 | -0.13 | 0.11 | -0.03 | -0.17 | 0.08 | -0.12 | -0.11 | -0.03 | 0.02 | 0 | 0.20 |
| NTproANP | -0.23 | -0.27 | -0.12 | -0.18 | 0.15 | -0.05 | -0.27 | -0.07 | 0.23 | 0.28 | -0.10 | -0.13 | 0.18 |
| NTproBNP | 0.05 | 0.01 | -0.02 | 0.02 | 0.07 | 0.01 | 0.07 | 0.16 | 0.09 | 0.07 | 0.09 | -0.02 | -0.23 |
| OPG | -0.17 | -0.18 | -0.02 | -0.14 | -0.03 | -0.04 | -0.27 | -0.00 | -0.05 | 0.19 | -0.02 | 0.02 | 0.04 |
| OPN | 0.05 | 0.09 | 0.10 | 0.02 | -0.28 | 0.03 | 0.02 | 0.11 | -0.05 | 0.06 | 0.14 | 0.04 | 0.18 |
| p Selectin | -0.04 | -0.06 | -0.03 | -0.01 | -0.07 | 0.09 | -0.10 | -0.02 | 0.07 | 0.02 | -0.07 | 0.06 | -0.09 |
| PAI1 | -0.00 | -0.03 | -0.02 | 0.03 | -0.10 | 0.03 | 0.08 | -0.16 | -0.11 | -0.11 | 0.12 | 0.06 | 0.28 |
| Pentraxin 3 | 0.43 | 0.41 | -0.01 | 0.32 | -0.42 | -0.23 | 0.28 | 0.11 | -0.07 | -0.23 | 0.32 | 0.09 | -0.17 |
| Renin | -0.20 | 0.02 | 0.37 | -0.05 | 0.21 | 0.35 | 0.09 | -0.02 | 0.02 | -0.06 | 0.08 | 0.40 | -0.18 |
| ST2 | 0.32 | 0.35 | 0.08 | 0.29 | -0.15 | 0.11 | 0.36 | 0.11 | -0.25 | -0.40 | 0.28 | 0.43 | 0.08 |
| Syndecan 1 | -0.17 | -0.10 | 0.06 | -0.22 | -0.05 | -0.12 | -0.29 | -0.04 | 0.03 | 0.24 | 0.01 | 0.03 | 0.07 |
| Syndecan 4 | -0.24 | -0.14 | 0.15 | -0.03 | -0.12 | -0.11 | -0.23 | -0.22 | -0.05 | -0.02 | 0.20 | 0.05 | 0.04 |
| KIM1 | -0.08 | 0.09 | 0.31 | -0.22 | -0.01 | 0.28 | -0.04 | -0.06 | 0.36 | 0.17 | 0.19 | -0.04 | -0.19 |
| TIMP1 | 0.13 | 0.12 | -0.01 | 0.21 | -0.06 | -0.14 | 0.25 | 0.26 | -0.29 | 0.09 | 0.01 | 0.04 | 0.20 |
| TIMP4 | -0.03 | -0.01 | 0.03 | 0.25 | -0.23 | -0.10 | -0.15 | -0.11 | -0.35 | 0.04 | -0.06 | 0.01 | 0.09 |
| TNFR1 | 0.15 | 0.15 | 0.05 | 0.34 | -0.42 | -0.01 | 0.13 | 0.17 | -0.22 | -0.08 | 0.14 | 0.29 | -0.15 |
| TNFR2 | 0.13 | 0.07 | -0.09 | 0.22 | -0.22 | -0.26 | 0.09 | -0.13 | -0.05 | -0.02 | 0.01 | 0.07 | 0.22 |
| TNF alpha | -0.09 | 0.05 | 0.25 | -0.05 | 0.10 | 0.14 | 0.01 | -0.20 | 0.00 | -0.02 | 0.32 | 0.19 | -0.30 |
| Tenascin C | 0.03 | -0.13 | -0.28 | 0.23 | -0.06 | -0.03 | -0.14 | 0.02 | 0.06 | -0.02 | -0.11 | 0.05 | 0.01 |
| Troponin T | 0.24 | 0.17 | -0.14 | 0.17 | -0.01 | -0.04 | 0.15 | -0.02 | 0.06 | -0.15 | -0.04 | 0.23 | 0.12 |
| VEGFR1 | 0.15 | 0.15 | -0.02 | 0.23 | -0.24 | -0.20 | 0.05 | -0.01 | -0.09 | -0.12 | -0.02 | 0.05 | 0.03 |
| VEGFa | 0.13 | 0.13 | -0.02 | 0.02 | 0.01 | 0.02 | 0.07 | 0.00 | -0.32 | -0.29 | 0.43 | 0.21 | 0.05 |
| Leptin | -0.64 | -0.51 | 0.31 | -0.53 | 0.49 | 0.32 | -0.41 | -0.07 | 0.01 | 0.53 | -0.06 | -0.54 | -0.13 |

Data reported as Pearson’s correlation co-efficient (r) green background highlighted *p*-value less than ≤0.01, yellow background highlighted *p*-value less than ≤0.05

Supplementary Table 3. Significant Pearson’s correlation co-efficient for healthy and T2D participants

| Biomarker | T2D | | | | | | | | | | | | |  | Healthy | | | | | | | | | | | | |
| --- | --- | --- | --- | --- | --- | --- | --- | --- | --- | --- | --- | --- | --- | --- | --- | --- | --- | --- | --- | --- | --- | --- | --- | --- | --- | --- | --- |
|  | LV EDVi | LV massi | LV m:v | LV GLS | LV long PEDSR | LV circ PEDSR | LV GCS | LAVImax | LA EF | E?/e' | LGE | Global MPR | Mean AD |  | LV EDVi | LV massi | LV m:v | LV GLS | LV long PEDSR | LV circ PEDSR | LV GCS | LAVImax | LA EF | E/e' | LGE | Global MPR | Mean AD |
| Adiponectin |  |  |  |  |  |  |  |  |  |  |  |  |  |  |  |  |  |  |  |  |  |  |  |  |  |  |  |
| Angiopoietin |  |  |  |  |  |  |  |  |  |  |  |  |  |  |  |  |  |  |  |  |  |  |  |  |  |  |  |
| BNP |  |  |  |  |  |  |  |  |  |  |  |  |  |  |  |  |  |  |  |  |  |  |  |  |  |  |  |
| CRP BL |  |  |  |  |  |  |  |  |  |  |  |  |  |  |  |  |  |  |  |  |  |  |  |  |  |  |  |
| CHI3L |  |  |  |  |  |  |  |  |  |  |  |  |  |  |  |  |  |  |  |  |  |  |  |  |  |  |  |
| Cystatin C |  |  |  |  |  |  |  |  |  |  |  |  |  |  |  |  |  |  |  |  |  |  |  |  |  |  |  |
| Endoglin |  |  |  |  |  |  |  |  |  |  |  |  |  |  |  |  |  |  |  |  |  |  |  |  |  |  |  |
| Endostatin |  |  |  |  |  |  |  |  |  |  |  |  |  |  |  |  |  |  |  |  |  |  |  |  |  |  |  |
| ET1 |  |  |  |  |  |  |  |  |  |  |  |  |  |  |  |  |  |  |  |  |  |  |  |  |  |  |  |
| FABP3 |  |  |  |  |  |  |  |  |  |  |  |  |  |  |  |  |  |  |  |  |  |  |  |  |  |  |  |
| FABP4 |  |  |  |  |  |  |  |  |  |  |  |  |  |  |  |  |  |  |  |  |  |  |  |  |  |  |  |
| FGF21 |  |  |  |  |  |  |  |  |  |  |  |  |  |  |  |  |  |  |  |  |  |  |  |  |  |  |  |
| FGF23 |  |  |  |  |  |  |  |  |  |  |  |  |  |  |  |  |  |  |  |  |  |  |  |  |  |  |  |
| Fas |  |  |  |  |  |  |  |  |  |  |  |  |  |  |  |  |  |  |  |  |  |  |  |  |  |  |  |
| GDF15 |  |  |  |  |  |  |  |  |  |  |  |  |  |  |  |  |  |  |  |  |  |  |  |  |  |  |  |
| Galectin 3 |  |  |  |  |  |  |  |  |  |  |  |  |  |  |  |  |  |  |  |  |  |  |  |  |  |  |  |
| ICAM1 |  |  |  |  |  |  |  |  |  |  |  |  |  |  |  |  |  |  |  |  |  |  |  |  |  |  |  |
| IL1 beta |  |  |  |  |  |  |  |  |  |  |  |  |  |  |  |  |  |  |  |  |  |  |  |  |  |  |  |
| IL10 |  |  |  |  |  |  |  |  |  |  |  |  |  |  |  |  |  |  |  |  |  |  |  |  |  |  |  |
| IL6 |  |  |  |  |  |  |  |  |  |  |  |  |  |  |  |  |  |  |  |  |  |  |  |  |  |  |  |
| IL8 |  |  |  |  |  |  |  |  |  |  |  |  |  |  |  |  |  |  |  |  |  |  |  |  |  |  |  |
| NGAL |  |  |  |  |  |  |  |  |  |  |  |  |  |  |  |  |  |  |  |  |  |  |  |  |  |  |  |
| MMP12 |  |  |  |  |  |  |  |  |  |  |  |  |  |  |  |  |  |  |  |  |  |  |  |  |  |  |  |
| MMP2 |  |  |  |  |  |  |  |  |  |  |  |  |  |  |  |  |  |  |  |  |  |  |  |  |  |  |  |
| MMP3 |  |  |  |  |  |  |  |  |  |  |  |  |  |  |  |  |  |  |  |  |  |  |  |  |  |  |  |
| MMP7 |  |  |  |  |  |  |  |  |  |  |  |  |  |  |  |  |  |  |  |  |  |  |  |  |  |  |  |
| MMP8 |  |  |  |  |  |  |  |  |  |  |  |  |  |  |  |  |  |  |  |  |  |  |  |  |  |  |  |
| MMP9 |  |  |  |  |  |  |  |  |  |  |  |  |  |  |  |  |  |  |  |  |  |  |  |  |  |  |  |
| MPO |  |  |  |  |  |  |  |  |  |  |  |  |  |  |  |  |  |  |  |  |  |  |  |  |  |  |  |
| NTproANP |  |  |  |  |  |  |  |  |  |  |  |  |  |  |  |  |  |  |  |  |  |  |  |  |  |  |  |
| NTproBNP |  |  |  |  |  |  |  |  |  |  |  |  |  |  |  |  |  |  |  |  |  |  |  |  |  |  |  |
| OPG |  |  |  |  |  |  |  |  |  |  |  |  |  |  |  |  |  |  |  |  |  |  |  |  |  |  |  |
| OPN |  |  |  |  |  |  |  |  |  |  |  |  |  |  |  |  |  |  |  |  |  |  |  |  |  |  |  |
| p Selectin |  |  |  |  |  |  |  |  |  |  |  |  |  |  |  |  |  |  |  |  |  |  |  |  |  |  |  |
| PAI1 |  |  |  |  |  |  |  |  |  |  |  |  |  |  |  |  |  |  |  |  |  |  |  |  |  |  |  |
| **Pentraxin 3** |  |  |  |  |  |  |  |  |  |  |  |  |  |  |  |  |  |  |  |  |  |  |  |  |  |  |  |
| Renin |  |  |  |  |  |  |  |  |  |  |  |  |  |  |  |  |  |  |  |  |  |  |  |  |  |  |  |
| ST2 |  |  |  |  |  |  |  |  |  |  |  |  |  |  |  |  |  |  |  |  |  |  |  |  |  |  |  |
| Syndecan 1 |  |  |  |  |  |  |  |  |  |  |  |  |  |  |  |  |  |  |  |  |  |  |  |  |  |  |  |
| Syndecan 4 |  |  |  |  |  |  |  |  |  |  |  |  |  |  |  |  |  |  |  |  |  |  |  |  |  |  |  |
| KIM1 |  |  |  |  |  |  |  |  |  |  |  |  |  |  |  |  |  |  |  |  |  |  |  |  |  |  |  |
| TIMP1 |  |  |  |  |  |  |  |  |  |  |  |  |  |  |  |  |  |  |  |  |  |  |  |  |  |  |  |
| TIMP4 |  |  |  |  |  |  |  |  |  |  |  |  |  |  |  |  |  |  |  |  |  |  |  |  |  |  |  |
| TNFR1 |  |  |  |  |  |  |  |  |  |  |  |  |  |  |  |  |  |  |  |  |  |  |  |  |  |  |  |
| TNFR2 |  |  |  |  |  |  |  |  |  |  |  |  |  |  |  |  |  |  |  |  |  |  |  |  |  |  |  |
| TNF alpha |  |  |  |  |  |  |  |  |  |  |  |  |  |  |  |  |  |  |  |  |  |  |  |  |  |  |  |
| Tenascin C |  |  |  |  |  |  |  |  |  |  |  |  |  |  |  |  |  |  |  |  |  |  |  |  |  |  |  |
| Troponin T |  |  |  |  |  |  |  |  |  |  |  |  |  |  |  |  |  |  |  |  |  |  |  |  |  |  |  |
| VEGFR1 |  |  |  |  |  |  |  |  |  |  |  |  |  |  |  |  |  |  |  |  |  |  |  |  |  |  |  |
| VEGFa |  |  |  |  |  |  |  |  |  |  |  |  |  |  |  |  |  |  |  |  |  |  |  |  |  |  |  |
| **Leptin** |  |  |  |  |  |  |  |  |  |  |  |  |  |  |  |  |  |  |  |  |  |  |  |  |  |  |  |

Green background highlighted *p*-value less than ≤0.01, yellow background highlighted *p*-value less than ≤0.05. Bolded biomarkers represent those who have the same CMR correlate in each group.

Supplementary Table 4: Heat map of Pearson’s correlation co-efficients between CMR outcomes of interest and each biomarker

| Biomarkers | T2D | | | | | | | | | | | | | |  | | Healthy | | | | | | | | | | | | | |
| --- | --- | --- | --- | --- | --- | --- | --- | --- | --- | --- | --- | --- | --- | --- | --- | --- | --- | --- | --- | --- | --- | --- | --- | --- | --- | --- | --- | --- | --- | --- |
|  | LV EDVi | LV massi | LV m:v | LV GLS | LV long PEDSR | LV circ PEDSR | LV GCS | LAVImax | LA EF | E?/e' | LGE | Global MPR | Mean AD |  | | LV EDVi | | LV massi | LV m:v | LV GLS | LV long PEDSR | LV circ PEDSR | LV GCS | LAVImax | LA EF | E/e' | LGE | Global MPR | Mean AD |  |
| Adiponectin |  |  |  |  |  |  |  |  |  |  |  |  |  |  | |  | |  |  |  |  |  |  |  |  |  |  |  |  |  |
| Angiopoietin |  |  |  |  |  |  |  |  |  |  |  |  |  |  | |  | |  |  |  |  |  |  |  |  |  |  |  |  |  |
| BNP |  |  |  |  |  |  |  |  |  |  |  |  |  |  | |  | |  |  |  |  |  |  |  |  |  |  |  |  |  |
| CRP BL |  |  |  |  |  |  |  |  |  |  |  |  |  |  | |  | |  |  |  |  |  |  |  |  |  |  |  |  |  |
| CHI3L |  |  |  |  |  |  |  |  |  |  |  |  |  |  | |  | |  |  |  |  |  |  |  |  |  |  |  |  |  |
| Cystatin C |  |  |  |  |  |  |  |  |  |  |  |  |  |  | |  | |  |  |  |  |  |  |  |  |  |  |  |  |  |
| Endoglin |  |  |  |  |  |  |  |  |  |  |  |  |  |  | |  | |  |  |  |  |  |  |  |  |  |  |  |  |  |
| Endostatin |  |  |  |  |  |  |  |  |  |  |  |  |  |  | |  | |  |  |  |  |  |  |  |  |  |  |  |  |  |
| ET1 |  |  |  |  |  |  |  |  |  |  |  |  |  |  | |  | |  |  |  |  |  |  |  |  |  |  |  |  |  |
| FABP3 |  |  |  |  |  |  |  |  |  |  |  |  |  |  | |  | |  |  |  |  |  |  |  |  |  |  |  |  |  |
| FABP4 |  |  |  |  |  |  |  |  |  |  |  |  |  |  | |  | |  |  |  |  |  |  |  |  |  |  |  |  |  |
| FGF21 |  |  |  |  |  |  |  |  |  |  |  |  |  |  | |  | |  |  |  |  |  |  |  |  |  |  |  |  |  |
| FGF23 |  |  |  |  |  |  |  |  |  |  |  |  |  |  | |  | |  |  |  |  |  |  |  |  |  |  |  |  |  |
| Fas |  |  |  |  |  |  |  |  |  |  |  |  |  |  | |  | |  |  |  |  |  |  |  |  |  |  |  |  |  |
| GDF15 |  |  |  |  |  |  |  |  |  |  |  |  |  |  | |  | |  |  |  |  |  |  |  |  |  |  |  |  |  |
| Galectin 3 |  |  |  |  |  |  |  |  |  |  |  |  |  |  | |  | |  |  |  |  |  |  |  |  |  |  |  |  |  |
| ICAM1 |  |  |  |  |  |  |  |  |  |  |  |  |  |  | |  | |  |  |  |  |  |  |  |  |  |  |  |  |  |
| IL1 beta |  |  |  |  |  |  |  |  |  |  |  |  |  |  | |  | |  |  |  |  |  |  |  |  |  |  |  |  |  |
| IL10 |  |  |  |  |  |  |  |  |  |  |  |  |  |  | |  | |  |  |  |  |  |  |  |  |  |  |  |  |  |
| IL6 |  |  |  |  |  |  |  |  |  |  |  |  |  |  | |  | |  |  |  |  |  |  |  |  |  |  |  |  |  |
| IL8 |  |  |  |  |  |  |  |  |  |  |  |  |  |  | |  | |  |  |  |  |  |  |  |  |  |  |  |  |  |
| NGAL |  |  |  |  |  |  |  |  |  |  |  |  |  |  | |  | |  |  |  |  |  |  |  |  |  |  |  |  |  |
| MMP12 |  |  |  |  |  |  |  |  |  |  |  |  |  |  | |  | |  |  |  |  |  |  |  |  |  |  |  |  |  |
| MMP2 |  |  |  |  |  |  |  |  |  |  |  |  |  |  | |  | |  |  |  |  |  |  |  |  |  |  |  |  |  |
| MMP3 |  |  |  |  |  |  |  |  |  |  |  |  |  |  | |  | |  |  |  |  |  |  |  |  |  |  |  |  |  |
| MMP7 |  |  |  |  |  |  |  |  |  |  |  |  |  |  | |  | |  |  |  |  |  |  |  |  |  |  |  |  |  |
| MMP8 |  |  |  |  |  |  |  |  |  |  |  |  |  |  | |  | |  |  |  |  |  |  |  |  |  |  |  |  |  |
| MMP9 |  |  |  |  |  |  |  |  |  |  |  |  |  |  | |  | |  |  |  |  |  |  |  |  |  |  |  |  |  |
| MPO |  |  |  |  |  |  |  |  |  |  |  |  |  |  | |  | |  |  |  |  |  |  |  |  |  |  |  |  |  |
| NTproANP |  |  |  |  |  |  |  |  |  |  |  |  |  |  | |  | |  |  |  |  |  |  |  |  |  |  |  |  |  |
| NTproBNP |  |  |  |  |  |  |  |  |  |  |  |  |  |  | |  | |  |  |  |  |  |  |  |  |  |  |  |  |  |
| OPG |  |  |  |  |  |  |  |  |  |  |  |  |  |  | |  | |  |  |  |  |  |  |  |  |  |  |  |  |  |
| OPN |  |  |  |  |  |  |  |  |  |  |  |  |  |  | |  | |  |  |  |  |  |  |  |  |  |  |  |  |  |
| p Selectin |  |  |  |  |  |  |  |  |  |  |  |  |  |  | |  | |  |  |  |  |  |  |  |  |  |  |  |  |  |
| PAI1 |  |  |  |  |  |  |  |  |  |  |  |  |  |  | |  | |  |  |  |  |  |  |  |  |  |  |  |  |  |
| Pentraxin 3 |  |  |  |  |  |  |  |  |  |  |  |  |  |  | |  | |  |  |  |  |  |  |  |  |  |  |  |  |  |
| Renin |  |  |  |  |  |  |  |  |  |  |  |  |  |  | |  | |  |  |  |  |  |  |  |  |  |  |  |  |  |
| ST2 |  |  |  |  |  |  |  |  |  |  |  |  |  |  | |  | |  |  |  |  |  |  |  |  |  |  |  |  |  |
| Syndecan 1 |  |  |  |  |  |  |  |  |  |  |  |  |  |  | |  | |  |  |  |  |  |  |  |  |  |  |  |  |  |
| Syndecan 4 |  |  |  |  |  |  |  |  |  |  |  |  |  |  | |  | |  |  |  |  |  |  |  |  |  |  |  |  |  |
| KIM1 |  |  |  |  |  |  |  |  |  |  |  |  |  |  | |  | |  |  |  |  |  |  |  |  |  |  |  |  |  |
| TIMP1 |  |  |  |  |  |  |  |  |  |  |  |  |  |  | |  | |  |  |  |  |  |  |  |  |  |  |  |  |  |
| TIMP4 |  |  |  |  |  |  |  |  |  |  |  |  |  |  | |  | |  |  |  |  |  |  |  |  |  |  |  |  |  |
| TNFR1 |  |  |  |  |  |  |  |  |  |  |  |  |  |  | |  | |  |  |  |  |  |  |  |  |  |  |  |  |  |
| TNFR2 |  |  |  |  |  |  |  |  |  |  |  |  |  |  | |  | |  |  |  |  |  |  |  |  |  |  |  |  |  |
| TNF alpha |  |  |  |  |  |  |  |  |  |  |  |  |  |  | |  | |  |  |  |  |  |  |  |  |  |  |  |  |  |
| Tenascin C |  |  |  |  |  |  |  |  |  |  |  |  |  |  | |  | |  |  |  |  |  |  |  |  |  |  |  |  |  |
| Troponin T |  |  |  |  |  |  |  |  |  |  |  |  |  |  | |  | |  |  |  |  |  |  |  |  |  |  |  |  |  |
| VEGFR1 |  |  |  |  |  |  |  |  |  |  |  |  |  |  | |  | |  |  |  |  |  |  |  |  |  |  |  |  |  |
| VEGFa |  |  |  |  |  |  |  |  |  |  |  |  |  |  | |  | |  |  |  |  |  |  |  |  |  |  |  |  |  |
| Leptin |  |  |  |  |  |  |  |  |  |  |  |  |  |  | |  | |  |  |  |  |  |  |  |  |  |  |  |  |  |

| -0.65 | -0.60 | -0.55 | -0.50 | -0.45 | -0.40 | -0.35 | -0.30 | -0.25 | -0.20 | -0.15 | -0.10 | -0.05 | 0.00 | 0.05 | 0.10 | 0.15 | 0.20 | 0.25 | 0.30 | 0.35 | 0.40 | 0.45 | 0.50 | 0.55 |
| --- | --- | --- | --- | --- | --- | --- | --- | --- | --- | --- | --- | --- | --- | --- | --- | --- | --- | --- | --- | --- | --- | --- | --- | --- |
|  |  |  |  |  |  |  |  |  |  |  |  |  |  |  |  |  |  |  |  |  |  |  |  |  |

Pearson correlation Co-efficient

Supplementary Table 5: Variables with missing values

| Variable | LGE | E/e' | LAVImax | LA EF | LV GCS | Global MPR | Mean AD | Leptin |
| --- | --- | --- | --- | --- | --- | --- | --- | --- |
| Missing | 3 | 8 | 1 | 1 | 1 | 16 | 2 | 1 |

Supplementary Table 6: Within group changes pre- post intervention in variables of interest

| **Variable** | **Δ MRP**  **(n=24)** | ***P-value*** | **Δ Exercise**  **(n=22)** | ***P-value*** | **Δ Control**  **(n=30)** | ***P-value*** |
| --- | --- | --- | --- | --- | --- | --- |
| HbA1c (%) | -0.96 ± 0.95 | **<0.001** | -0.10 ± 0.52 | 0.360 | -0.09 ± 0.68 | 0.472 |
| Fasting Glucose | -1.80 ± 2.10 | **<0.001** | -0.67 ± 1.64 | 0.075 | -0.19 ± 2.11 | 0.632 |
| HOMA-IR | -7.45 ± 8.37 | **<0.001** | -2.25 ± 7.29 | 0.173 | 0.44 ± 8.85 | 0.786 |
| Weight (Kg) | -13.68 ± 4.81 | **<0.001** | -1.38 ± 1.55 | **<0.001** | -2.28 ± 3.73 | **0.002** |
| BMI (kgm^2^) | -4.71 ± 1.69 | **<0.001** | -0.44 ± 0.59 | **0.002** | -0.84 ± 1.41 | **0.003** |
| Total Fat mass (kg) | -9.70 ± 3.32 | **<0.001** | -0.67 ± 1.36 | 0.051 | -1.21 ± 2.87 | **0.046** |
| Total lean mass (kg) | -3.33 ± 21.33 | **<0.001** | -0.13 ± 1.68 | 0.753 | -0.41 ± 1.87 | 0.285 |
| Total visceral fat (kg) | -0.93 ± 0.47 | **<0.001** | -0.03 ± 0.29 | 0.644 | -0.06 ± 0.33 | 0.355 |
| Pancreatic fat (%) | -1.38 ± 2.24 | **0.046** | -0.22 ±1.12 | 0.502 | -0.57 ± 2.53 | 0.331 |
| Liver fat (%) | -7.78 ± 5.89 | **<0.001** | -0.74 ± 5.30 | 0.612 | -2.13 ± 5.03 | 0.073 |
| **Cardiac structure and function** | | | | | | |
| LV EDV (mL) | -1.49 ± 13.30 | 0.958 | 2.45 ± 15.37 | 0.462 | -0.37 ± 11.72 | 0.862 |
| LV Mass (g) | -5.56 ± 13.80 | 0.066 | 1.15 ± 12.60 | 0.673 | 0.90 ± 10.25 | 0.634 |
| LVmass:volume (g/mL)* | -0.04 (0.09) | **0.023** | -0.00 (0.07) | 0.158 | 0.02 (0.11) | 0.280 |
| LV GLS (+%) | -0.61 ± 2.57 | 0.271 | -0.23 ± 2.31 | 0.645 | -0.63 ± 2.09 | 0.112 |
| LV GCS (+%) | -1.32 ± 2.43 | **0.016** | -0.25 ± 2.08 | 0.584 | -0.57 ± 1.82 | 0.096 |
| LV LongPEDSR (s-1) | -0.063 ± 0.11 | **0.012** | -0.00 ± 0.21 | 0.976 | 0.01 ± 0.154 | 0.613 |
| LV CircPEDSR (s-1) | -0.05 ± 0.18 | 0.199 | -0.10 ± 0.14 | **0.002** | -0.07 ± 0.14 | **0.008** |
| LAVmax (mL)* | -1.30 (23.97) | 0.903 | -3.59 (15.10) | 0.527 | 0.71 (14.68) | 0.347 |
| LA EF (%) | 0.33 ± 7.02 | 0.823 | -1.19 ± 8.55 | 0.522 | -0.57 ± 1.82 | 0.096 |
| LV EF (%) | 4.54 ± 5.45 | 0.001 | 0.79 ± 6.74 | 0.588 | -1.42 ± 6.27 | 0.225 |
| Mean Ao Distens (mmHg^-1^x10^-3^) | 0.90 ± 1.18 | **0.001** | 0.55 ± 3.11 | 0.427 | 0.51 ± 1.85 | **0.153** |
| MPR | 0.18 ± 1.23 | 0.550 | 0.10 ± 1.20 | 0.735 | 0.48 ± 1.18 | 0.062 |
| LGE n (%)** | 5 (20.83) |  | 0 (0.00) | 0.46 | 2 (6.67) | 0.897 |
| Average E/e'* | -0.55 (3.32) | 0.204 | -0.27 (4.05) | 0.267 | -0.03 (2.53) | 0.696 |

Abbreviations: LV EDV = Left Ventricular End Diastolic Volume, LV Mass = Left Ventricular Mass, LV mass:volume = Left Ventricular mass to volume ratio, LV GLS = Left Ventricular global longitudinal strain, LV LongPEDSR = Left Ventricular Longitudinal Peak Early Diastolic Strain rate, LV CircPEDSR = Left ventricular Circumferential Peak Early Diastolic Strain Rate, LV GCS = Left ventricular global circumferential strain, LAVImax = Left atrial maximum volume, LA EF = Left Atrial Ejection Fraction, LV EF = Left ventricular ejection fraction, Mean A Distens = Mean aortic distensibility, MPR= Myocardial perfusion reserve, LGE = Late Gadolinium Enhancement present. *P*-value reported for paired t-test, *Wilcoxon-signed-rank test. ** Chi squared or fishers exact where count is <5 in a cell. Data are reported as mean ± standard deviation, count (percent) or median (interquartile range). Bold font highlights statistically significant difference with significance level of 95%.

Supplementary Table 7 | Repeated measures analysis with reported estimated mean change in fibro-inflammatory markers

| **Δ biomarker** | **Δ MRP unadjusted model 1** | | | **Δ MRP adjusted model 2** | | | **Δ MRP adjusted model 3** | | |
| --- | --- | --- | --- | --- | --- | --- | --- | --- | --- |
|  | **EMM** | **95%CI** | ***P-*value** | **EMM** | **95%CI** | ***P-*value** | **EMM** | **95%CI** | ***P-*value** |
| Inflammatory |  |  |  |  |  |  |  |  |  |
| Leptin | -0.37 | -0.45, 0.29 | <0.001 | -0.37 | -0.42, 0.32 | <0*.*001 | -0*.*37 | -0*.*45, -0*.*29 | <0*.*001 |
| Adiponectin | 0.11 | 0.01, 0.21 | 0.030 | 0.11 | 0.02, 0.19 | 0*.*015 | 0*.*11 | 0*.*02, 0*.*12 | 0*.*022 |
| FABP4 | 0.11 | 0.03, 0.19 | 0.008 | 0.11 | 0.04, 0.18 | 0*.*005 | 0*.*11 | 0*.*03, 0*.*19 | 0*.*010 |
| NGAL | 0.14 | 0.07, 0.20 | <0.001 | 0.14 | 0.07, 0.20 | <0*.*001 | 0*.*14 | 0*.*70, 0*.*20 | <0*.*001 |
| OPN | 0.13 | 0.06, 0.21 | 0.002 | 0.13 | 0.05, 0.21 | 0*.*002 | 0*.*13 | 0*.*06, 0*.*20 | 0*.*001 |
| Pentraxin 3 | 0.42 | 0.29, 0.55 | <0.001 | 0.42 | 0.31, 0.53 | <0*.*001 | 0*.*42 | 0*.*29, 0*.*55 | <0*.*001 |
| Fibrotic |  |  |  |  |  |  |  |  |  |
| MMP2 | 0.06 | 0.02, 0.11 | 0.009 | 0.06 | 0.02, 0.11 | 0*.*010 | 0*.*06 | 0*.*02, 0*.*11 | 0*.*008 |
| MMP8 | 0.09 | 0.01, 0.17 | 0.023 | 0.09 | 0.01, 0.17 | 0*.*023 | 0*.*09 | 0*.*01, 0*.*17 | 0*.*023 |
| MMP9 | 0.18 | 0.05, 0.31 | 0.011 | 0.18 | 0.04, 0.31 | 0*.*012 | 0*.*18 | 0*.*04, 0*.*31 | 0*.*015 |
| CHI3L1 | -0.11 | -0.21, -0.01 | 0.038 | -0.11 | -0.21, -0.01 | 0*.*036 | -0*.*11 | -0*.*21, -0*.*00 | 0*.*044 |
| PAI1 | -0.15 | -0.25, -0.04 | 0.008 | -0.15 | -0.26, -0.04 | 0*.*011 | -0*.*15 | -0*.*26, -0*.*04 | 0*.*009 |
| Endothelial function |  |  |  |  |  |  |  |  |  |
| Endostatin | 0.07 | 0.01, 0.14 | 0.027 | 0.07 | 0.01, 0.14 | 0*.*032 | 0*.*07 | 0*.*01, 0*.*14 | 0*.*024 |
| ET1 | 0.06 | 0.00, 0.12 | 0.039 | 0.06 | 0.01, 0.12 | 0*.*036 | 0*.*06 | 0*.*00, 0*.*12 | 0*.*041 |
| VEGFR1 | 0.15 | 0.06, 0.23 | 0.003 | 0.15 | 0.06, 0.23 | 0*.*003 | 0*.*15 | 0*.*05, 0*.*24 | 0*.*004 |
| Atrial stress/stretch |  |  |  |  |  |  |  |  |  |
| NTproANP | 0.14 | 0.04, 0.25 | 0.008 | 0.14 | 0.04,0.25 | 0*.*010 | 0*.*14 | 0*.*04, 0*.*25 | 0*.*010 |
| **Δ biomarker** | **Δ Exercise unadjusted model 1** | | | **Δ Exercise adjusted model 2** | | | **Δ Exercise adjusted model 3** | | |
|  | **EMM** | **95%CI** | ***P-*value** | **EMM** | **95%CI** | ***P-*value** | **EMM** | **95%CI** | ***P-*value** |
| Fibrotic |  |  |  |  |  |  |  |  |  |
| MMP8 | 0.14 | 0.01, 0.26 | 0.037 | 0.14 | 0.02, 0.26 | 0*.*030 | 0*.*14 | 0*.*00, 0*.*27 | 0*.*047 |
| MMP9 | 0.21 | 0.05, 0.37 | 0.013 | 0.21 | 0.05, 0.36 | 0*.*011 | 0*.*21 | 0*.*05, 0*.*37 | 0*.*014 |

All data log10. Abbreviations: EMM = estimated marginal means, 95%CI: 95% Confidence Interval. Model 1: Unadjusted, Model 2: Adjusted for change in weight and baseline weight in kilograms, Model 3: Change in HbA1c and baseline HbA1c. Bonferroni correction applied for all tests to account for multiple comparisons (observed uncorrected p-value multiplied by the number of comparisons).

Supplementary Table 8a-b| The 30 most informative components of horizontal and vertical axis for change.

| Attribute | Horizontal |  | Attribute | Vertical |
| --- | --- | --- | --- | --- |
| BMI (kg/m2) | 0*.*3013 |  | MMP9 | -0.3246 |
| Weight (kg) | 0.2865 |  | Pentraxin 3 | -0.2932 |
| FABP4 | 0.2723 |  | VEGFR1 | -0.2546 |
| HbA1c (%) | 0.2654 |  | MMP8 | -0.2356 |
| Adiponectin | -0.2480 |  | LV EF (%) | 0.2299 |
| GDF15 | 0.2089 |  | LGE present (y/n) | -0.2204 |
| Fasting glucose (mmol/L) | 0.2080 |  | Syndecan 4 | -0.2090 |
| Average E/e' | 0.2003 |  | FABP4 | -0.2032 |
| LV mass/volume (g/ml) | 0.1902 |  | Fasting glucose (mmol/L) | 0.1901 |
| Tenascin C | -0.1804 |  | LV GCS (%) | -0.1747 |
| HOMA IR | 0.1614 |  | LV GLS (%) | -0.1690 |
| MMP8 | 0.1575 |  | HOMA IR | 0.1662 |
| MMP2 | -0.1478 |  | Galectin 3 | -0.1510 |
| MMP3 | -0.1472 |  | Mean aortic distensibility | -0.1448 |
| Mean aortic distensibility | -0.1444 |  | Angiopoietin 2 | -0.1440 |
| Renin | 0.1388 |  | IL1 beta | -0.1403 |
| Leptin | 0.1386 |  | LV ESV (mL) | -0.1264 |
| Global MPR | -0.1381 |  | NGAL | -0.1246 |
| TNF alpha | 0.1312 |  | FGF23 | -0.1244 |
| MMP7 | 0.1299 |  | NTproANP | -0.1227 |
| IL6 | 0.1184 |  | HbA1c (%) | 0.1223 |
| IL8 | 0.1175 |  | LV long PEDSR (1/s) | 0.1221 |
| LV circ PEDSR (1/s) | -0.1162 |  | Weight (kg) | 0.1147 |
| TNFR1 | 0.1160 |  | IL8 | -0.1140 |
| LV GLS (%) | 0.1114 |  | BMI (kg/m2) | 0.1101 |
| LV mass (g) | 0.1065 |  | Leptin | 0.1086 |
| VEGFa | 0.1050 |  | p Selectin | 0.1036 |
| Cystatin C | -0.1023 |  | Global MPR | -0.0996 |
| MMP12 | -0*.*0938 |  | GDF15 | -0.0993 |
| PAI1 | 0*.*0913 |  | TIMP1 | 0.0884 |

All fibro-inflammatory biomarkers are Log10. The blue background highlights the attributes observed in both lists.

## 1.4 Figures

**Figure S1: 3D visualisation in space of demographic and CMR outcomes**


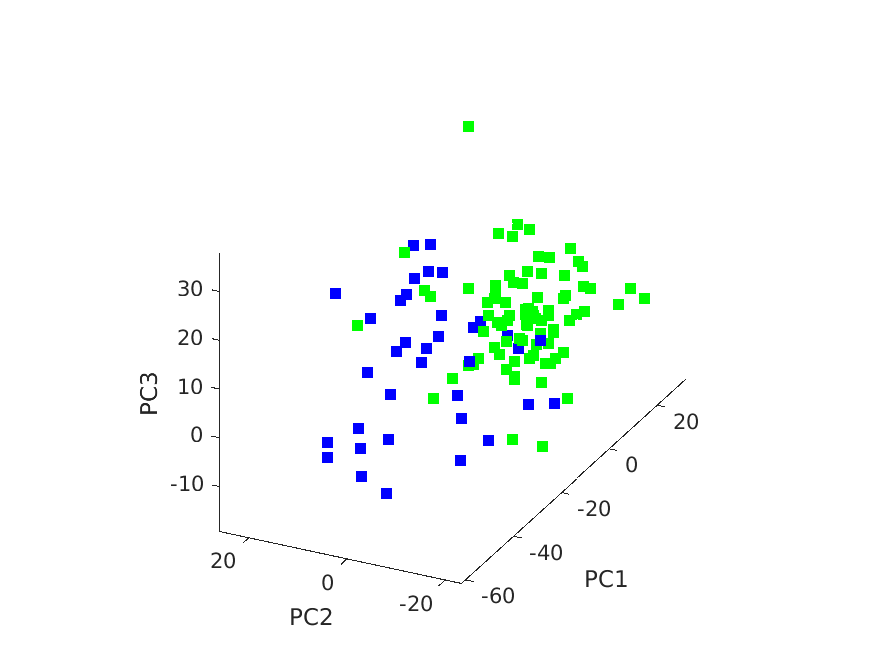


Blue data points: Healthy Volunteers (controls). Green data points: T2D (cases)

**Figure S2: 3D visualisation in space of demographic and fibro-inflammatory biomarkers**


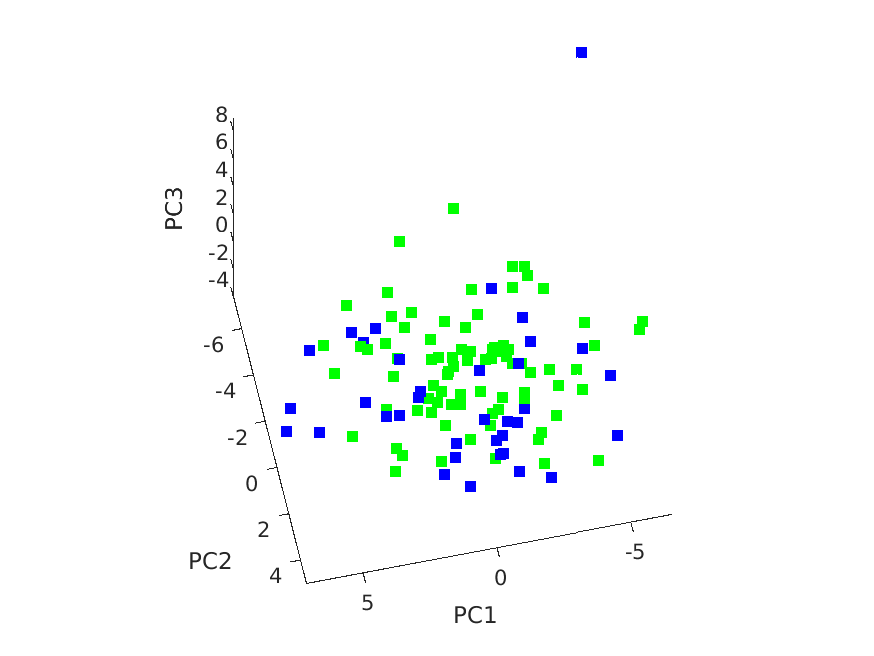


Blue data points: Healthy Volunteers (controls). Green data points: T2D (cases)

**Figure S3: 3D visualisation of four groups of patients in the space of the first three principal components calculated for demographic, CMR outcome and fibro-inflammatory biomarker variables**


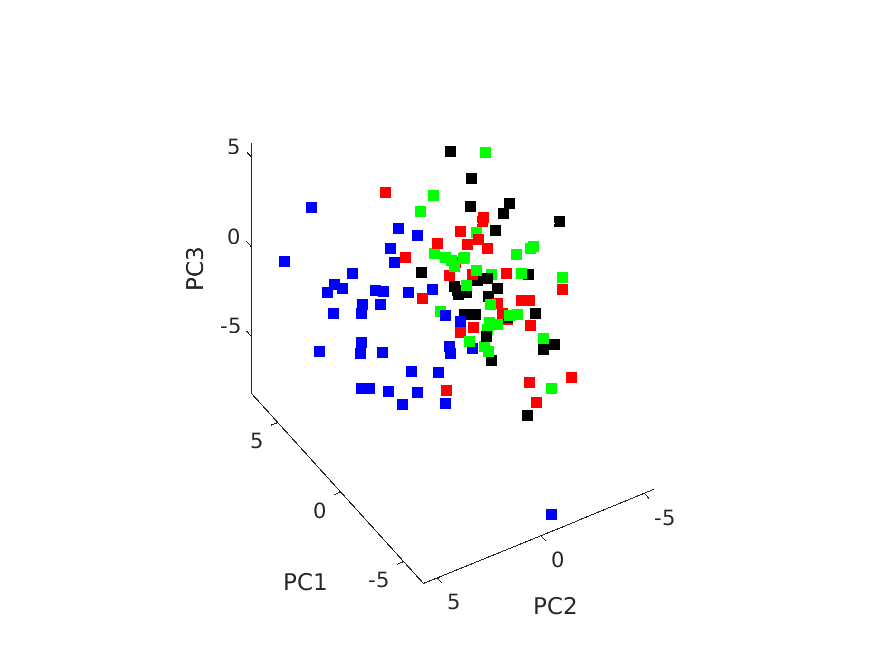


Blue = healthy volunteer, Green = T2D randomised to control, Red = T2D randomised to Exercise, Black = T2D randomised to MRP. All data taken from baseline visit.

## Description of search of plane of changes

There are three groups of participants: $H$ is group of healthy volunteers, $B$ is group of all participants with T2D before care, and $A$ is group of all participants with T2D after care. Central point of each group can be calculated as

$$h_{c}=\frac{1}{\left| H \right|}\sum_{x\in H} x,b_{c}=\frac{1}{\left| B \right|}\sum_{x\in B} x, a_{c}=\frac{1}{\left| A \right|}\sum_{x\in A} x,$$

where $\left| S \right|$ is number of participants in group $S$. It is clear that $\left| A \right|=\left| B \right|$. To find plane which contains points $h_{c}, b_{c}, a_{c}$ let us calculate two vectors: $w_{1}=a_{c}-h_{c}$ and $w_{2}=b_{c}-h_{c}$. To extract basis from these set of vectors we firstly normalise vector $w_{1}$ to unit length:

$$v_{1}=\frac{w_{1}}{\left\| w_{1} \right\|},$$

where $\left\| w_{1} \right\|$ is length of vector $w_{1}$. Now we subtract from vector $w_{2}$ projection of this vector onto vector $v_{1}$:

$$x=w_{2}-\left( w_{2},v_{1} \right)v_{1},$$

where $\left( w_{2},v_{1} \right)$ is dot product of vectors $w_{2}$ and $v_{1}$. The final step is normalisation of the last vector:

$$v_{2}=\frac{x}{\left\| x \right\|}.$$

Vectors $v_{1}$ and $v_{2}$ are basis vectors of the target plane.

References

1. Gulsin GS, Brady EM, Swarbrick DJ, et al. Rationale, design and study protocol of the randomised controlled trial: Diabetes Interventional Assessment of Slimming or Training tO Lessen Inconspicuous Cardiovascular Dysfunction (the DIASTOLIC study). *BMJ Open* 2019; **9**(3): e023207.

2. NICE. Natioanl Institute for Health and Care Excellence: Type 2 diabetes in adults: management [NG28]. <https://www.nice.org.uk/guidance/ng28>; 2015.

3. Tang H, Panemangalore R, Yarde M, Zhang L, Cvijic ME. 384-Well Multiplexed Luminex Cytokine Assays for Lead Optimization. *Journal of Biomolecular Screening* 2016; **21**(6): 548-55.

4. Khan JN, Wilmot EG, Leggate M, et al. Subclinical diastolic dysfunction in young adults with Type 2 diabetes mellitus: a multiparametric contrast-enhanced cardiovascular magnetic resonance pilot study assessing potential mechanisms. *Eur Heart J Cardiovasc Imaging* 2014; **15**(11): 1263-9.
